# Ballistic diffusion fronts in biomolecular condensates

**DOI:** 10.1038/s41565-025-01941-0

**Published:** 2025-06-06

**Authors:** Weixiang Chen, Brigitta Dúzs, Pablo G. Argudo, Sebastian V. Bauer, Wei Liu, Avik Samanta, Sapun H. Parekh, Mischa Bonn, Andreas Walther

**Affiliations:** 1 Life-Like Materials and Systems, Department of Chemistry, University of Mainz, Mainz, Germany; 2 Department of Molecular Spectroscopy, Max Planck Institute for Polymer Research, Mainz, Germany; 3 Department of Biomedical Engineering, University of Texas at Austin, Austin, TX USA; 4 Present Address: Department of Physical Chemistry, University of Córdoba, Córdoba, Spain; 5 Present Address: Department of Chemistry, Indian Institute of Technology Kharagpur, West Bengal, India

**Keywords:** Supramolecular chemistry, Biological physics, DNA nanotechnology, Condensed-matter physics

## Abstract

Biomolecular condensates in cells compartmentalize vital processes by enriching molecules through molecular recognition. However, it remains elusive how transport occurs in biomolecular condensates and how it relates to their dynamic and/or viscoelastic state. We show that the transport of molecules in DNA model condensates does not follow classical Fickian diffusion, which has a blurry front with a square root of time dependence. By contrast, we identify a new type of transport with an ultrasharp front that propagates linearly with time. Our data reveal that this ultrasharp ballistic diffusion front originates from molecular recognition and an arrested-to-dynamic transition in the condensate properties. This diffusion mechanism is the result of intertwining chemical kinetics and condensate dynamics on transport in biomolecular condensates. We believe that our understanding will help to better explain and tune the dynamics and properties in synthetic condensate systems and for biological functions.

## Abstract (web summary)

Adding short, complementary oligonucleotides to single-stranded DNA condensates creates a concentrated, linearly propagating, sharp diffusion front that contradicts the fuzzy concentration gradients and nonlinear kinetics typical of Fickian diffusion.

## Main

Biomolecular condensates formed by the liquid–liquid phase separation of nucleic acids^1,2^ and proteins^3,4^ in cells serve as signalling and reaction hubs^5,6^, and gate accessibility, for instance, to the nucleus^7^. The transfer of concepts from polymer science has greatly advanced the understanding of condensate formation, stabilization and their physicochemical properties^8–16^.

Despite this progress, a mechanistic understanding of how macromolecules are transported into and within condensates remains limited. Biomolecular condensates possess complex viscoelastic properties and are typically classified as liquid like or solid like^17–19^. However, the functional relevance of the solid-like state beyond regulating the mechanical properties and arresting dynamics is poorly understood^5,20^. In a wider content, in the crowded and heterogeneous environment of cells and condensates with complex viscoelastic properties^21–24^, transport may deviate from classical diffusion and be influenced by complex relaxation dynamics^25^ or specific interactions^26,27^, leading to an anomalous diffusion behaviour^7,28^. To dissect these intricacies, minimalistic synthetic model systems offer powerful platforms to study the behaviour in well-controlled settings^29,30^.

In this study, we report on the observation of a new diffusion behaviour during the uptake of macromolecules into biomolecular condensates driven by molecular recognition. Our findings reveal this unprecedented diffusion mechanism on the addition of a short oligonucleotide, termed ‘invader’, to DNA condensates composed of long single-stranded DNA (ssDNA). The invader selectively binds to complementary domains in the DNA condensate. During its uptake, a sharp diffusion front is formed with an accumulation of a high concentration of the invader at this front, which propagates with a linear time dependence. This contradicts the commonly observed fuzzy concentration gradients and nonlinear propagation kinetics described by Fickian diffusion, which widely applies to many diffusion processes in solutions. We quantify, model and extrapolate the multiscale non-equilibrium nature of this new diffusion mode and distinguish it from the current understanding of diffusion and transport in condensates. We discuss the generalization of the findings based on the physicochemical interplay between condensate dynamics, molecular recognition and chemical kinetics.

## Observation of ballistic diffusion fronts

**Extended Data Fig. 1:**
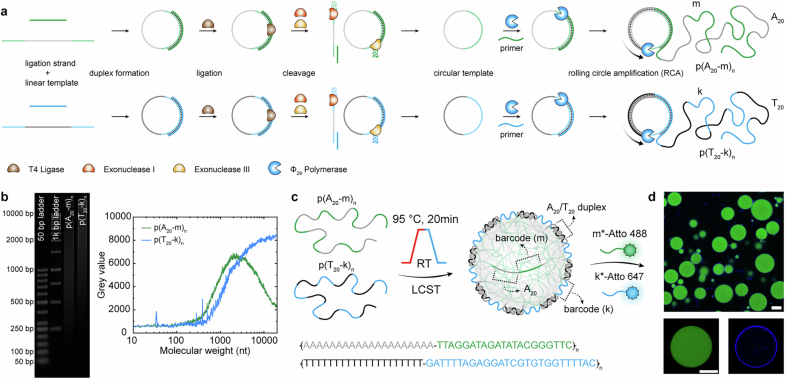
Synthesis of ssDNA polymers and preparation of DNA condensates. **a**, Schematic representation for the enzymatic synthesis of circular template by T4 Ligase and subsequent removal of non-circular strands by Exonuclease I and Exonuclease III. The circular template is then used for the enzymatic synthesis of DNA polymers (p(A_20_-m)_n_ and p(T_20_-k)_n_) by rolling circle amplification (RCA) using Φ_29_ DNA polymerase and a primer. **b**, Background-subtracted gel electrophoresis image of p(A_20_-m)_n_ and p(T_20_-k)_n_ after thermal cleavage at 95 °C for 30 min in total. Quantification of chain length of p(A_20_-m)_n_ and p(T_20_-k)_n_ is shown in the right plot. The molecular weight is reported as nucleotide number (nt), corresponding to double of the calibrated base pair number (bp). Average repeats of p(A_20_-m)_**74**_ can be calculated. p(T_20_-k)_n_ has a tailing to higher molecular weights (n > 90). This material is used for preparing the DNA condensates. **c**, Scheme for the preparation of DNA condensates by a heating ramp applied to a solution of 0.5 g/L of p(A_20_-m)_n_, and 0.0625 g/L p(T_20_-k)_n_ incubated in TE buffer containing 50 mM MgCl_2_ at 95 °C for 20 min, followed by cooling to room temperature. The heating and cooling rate are both 3 °C/s. The formed condensates are composed of a p(A_20_-m)_n_ core and a p(T_20_-k)_n_ shell, that can be individually addressed by the complementary strands (m* and k*) conjugated with fluorophores. Details can be found in our preceding publications^31,33^. **d**, Representative CLSM image showing the resulting DNA condensates with the bottom images showing the respectively addressed p(A_20_-m)_n_ core by m*-Atto488 (left, green channel) and p(T_20_-k)_n_ shell by k*-Atto647 (right, blue channel). Note the thin blue shell. Gel source data is provided. The scale bars are all 10 μm. Source data

We prepared DNA condensates by the temperature-induced coacervation of two long ssDNA copolymers, p(A_20_-m)_*n*_ and p(T_20_-k)_*n*_ in a TE buffer at 50 mM of MgCl_2_ (*n*, repeating units; Extended Data Fig. 1)^31–33^. A_20_ and T_20_ are homorepeats of adenine- and thymine-containing nucleotides, respectively, whereas m and k are DNA barcode sequences that can bind complementary oligonucleotides (Extended Data Fig. 1a). Briefly, on heating a p(A_20_-m)_*n*_/p(T_20_-k)_*n*_ mixture, p(A_20_-m)_*n*_ undergoes selective temperature-induced liquid–liquid phase separation and forms spherical condensates above its cloud point temperature (~42 °C)^31^, whereas p(T_20_-k)_*n*_ remains in solution. During cooling, p(T_20_-k)_*n*_ localizes at the periphery of p(A_20_-m)_*n*_ condensates by A_20_/T_20_ hybridization and forms a thin and permeable shell (Extended Data Fig. 1c)^32,33^. This process results in micrometre-sized core–shell condensates with addressable barcodes in the core (m) and shell (k). The shells are typically labelled at the k barcodes with fluorescent ssDNA (k*-dye) for visualization (Extended Data Fig. 1d)^31,32^. The m barcodes in the core provide recognition sites for the diffusing molecules (invaders, m*) and mimic molecular recognition in biological systems.

**Fig. 1:**
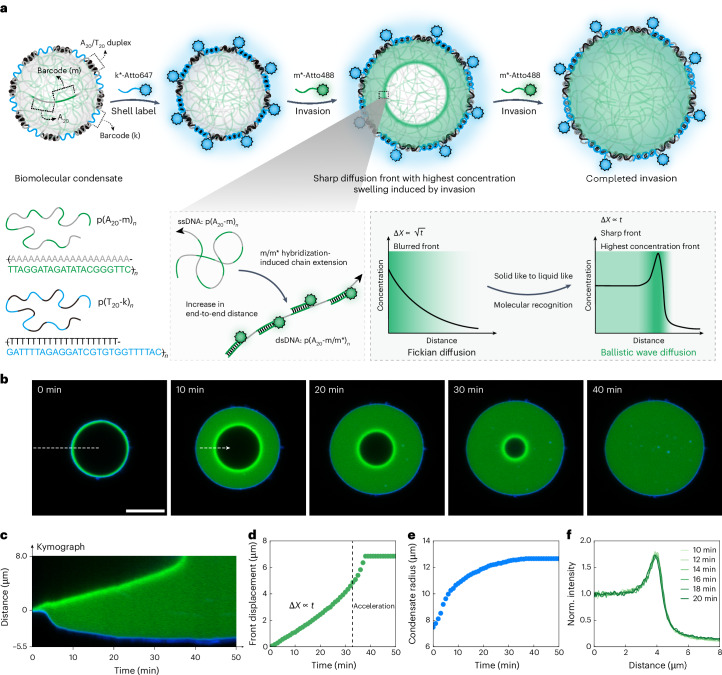
Not Fickian but ballistic wave diffusion with a high-intensity zone occurs during invasion of ssDNA invaders into DNA condensates. **a**, Scheme of ballistic wave diffusion in DNA condensates, whereby invaders m* bind to m barcodes in the DNA condensates, inducing a structural and conformational transition from ssDNA to dsDNA. Differences between classical Fickian diffusion and ballistic wave diffusion identified in this study. **b**, Time-series CLSM images showing the invasion process of invaders (m*-Atto488, 2 μM; green) into a DNA condensate (shell labelled by k*-Atto647; blue) at 50 mM of MgCl_2_. The ballistic wave diffusion process has been consistently reproduced in 20 individual concentration-dependent experiments discussed below as well as in other experiments throughout this research. **c**, Kymograph (space–time plot) of the invasion process along the white dashed line in **b**. **d**, Front displacement during invasion is linear with time. Acceleration happens at the end stage (noted with the dashed line) because of the interference between diffusion fronts from all the radial directions. **e**, Radius of DNA condensate during invasion. **f**, Cross-sectional line profiles taken at various times along the white dashed arrow in **b**. The distances are rescaled to the maximum intensity of the front to highlight the self-similar shape during invasion (Supplementary Video 1). Scale bar, 10 μm. Source data

**Extended Data Fig. 2:**
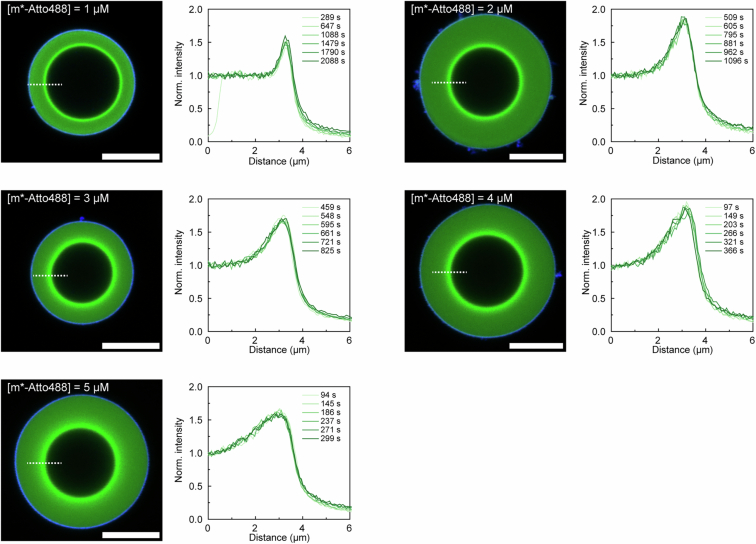
Stable diffusion fronts at individual concentrations of invader m*. Representative CLSM images showing ballistic wave diffusion at different invader concentrations ([m*-Atto488]) at 50 mM MgCl_2_. Time-dependent cross-sectional line profiles along the white dashed lines in each CLSM image are shown to the right of the corresponding CLSM images. The line profiles are rescaled on the distance axis to the maximum intensity of the front. As can be seen from the line profile analysis, the front is self-similar and always has a stable shape over time, indicating balanced kinetics at the front. 0.25 μM k*-Atto647 was added for the labeling of the shell (blue channel). See also Supplementary Video 1 for a kymograph. Scale bars for all are 10 μm. Source data

Surprisingly, the uptake of invaders (m*-Atto488), which specifically bind to the m barcodes inside the condensate, results in a sharp, high-intensity diffusion front (Fig. 1a,b and Supplementary Video 1). The front propagates linearly with time, that is, Δ*X* (front displacement) ∝ *t* (time) (Fig. 1c,d). This represents a ballistic behaviour (constant velocity) of the macroscopic diffusion front. Considerable swelling occurs in the invaded region to a final swelling ratio of about fourfold in volume (Fig. 1b,e). The high-intensity front arises from the spatial accumulation of the invaders and is stable in shape during propagation (Fig. 1f and Extended Data Fig. 2). This propagating steady state indicates the balanced kinetic processes of invader binding and condensate swelling at the front interface. This behaviour is fundamentally different from classical Fickian diffusion, which exhibits unavoidable concentration gradients and a power-law relationship of the front propagation with time, that is, Δ*X* ∝ *t*^1/2^ (Fig. 1a).

**Extended Data Fig. 3:**
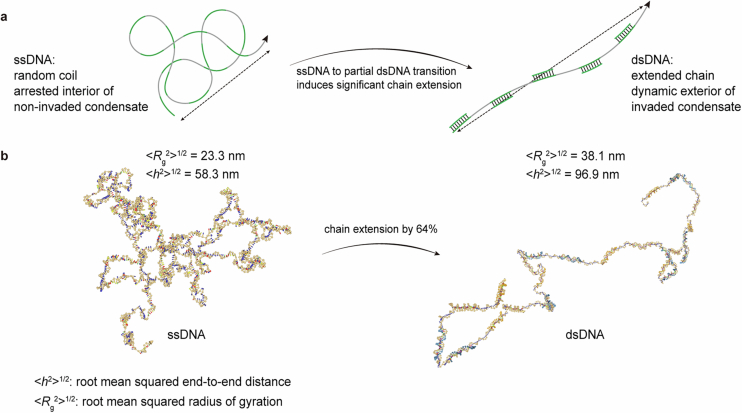
Molecular Dynamics (MD) simulations of the DNA chain extension induced by ssDNA to partial dsDNA transition upon hybridization of the invader m* to p(A_20_-m)_n_, leading to p(A_20_-m/m*)_n._ **a**, Schematic representation for the DNA chain extension after hybridization. **b**, Representative chain conformation of ssDNA and dsDNA obtained from oxDNA MD simulations. The root mean squared end-to-end distance ( < *h*^2^ > ^1/2^) and root mean squared radius of gyration ( < *R*_g_^2^ > ^1/2^) for ssDNA (p(A_20_-m)_n_) and dsDNA (p(A_20_-m/m*)_n_) are calculated over a simulation sample size of *N* = 20000. The total length of the strand is 1025 nt.

The sharp diffusion front is a non-equilibrium steady-state interface, where DNA chain extension occurs because m barcodes of the p(A_20_-m)_*n*_ ssDNA are converted to m/m* double-stranded DNA (dsDNA) segments. Molecular dynamics simulations using oxDNA yield a 64% increase in the radius of gyration from p(A_20_-m)_*n*_ to p(A_20_-m/m*)_*n*_ (Extended Data Fig. 3 and Supplementary Methods 1)^34–36^. An acceleration of the front propagation is visible in the final invasion stage, probably caused by the interference of individual diffusion fronts and the smaller surface area once the fronts start to share the same non-invaded centre. The process loosely resembles ‘case II diffusion’^37,38^ that describes the diffusion of solvents with sharp fronts into specific thermoplastics. However, despite a phenomenological similarity, case II diffusion in thermoplastics cannot account for a high-intensity front; critically, the origin and underlying physical principles are profoundly different. Condensates, unlike thermoplastics, are solvent-rich entities, and we elucidate that molecular recognition plays a key role—a specific property inherent to biomacromolecules and responsible for condensate formation in cells. We term this new molecular-recognition-enabled frontal diffusion mechanism with a high-intensity invader front as ‘ballistic wave diffusion’, which satisfies the condition of Δ*X* ∝ *t*.

## Mechanistic details of ballistic wave diffusion

**Fig. 2:**
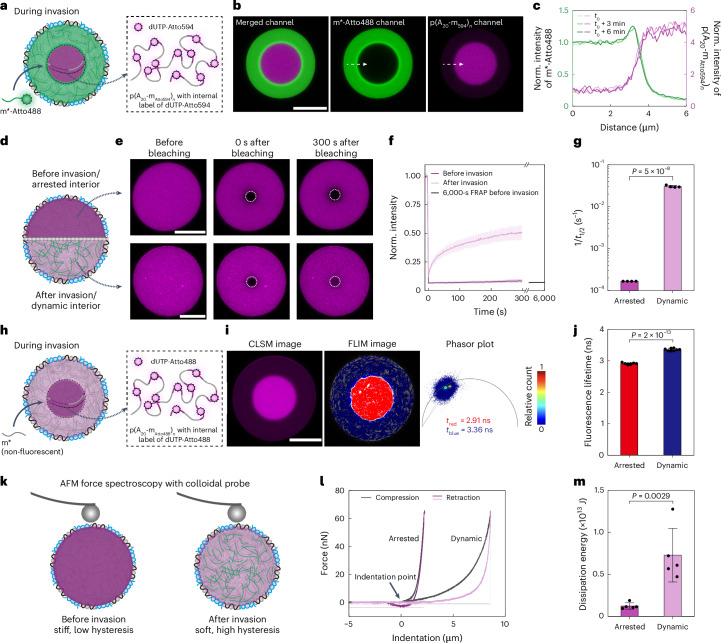
Changes in condensate dynamical and mechanical properties during ballistic wave diffusion. **a**, Scheme of a DNA condensate with in-chain fluorophores (p(A_20_-m_Atto594_)_*n*_; magenta) during the invasion of m*-Atto488 (green). **b**, CLSM images of a DNA condensate during invasion by 1 μM of m*-Atto488 at 50-mM MgCl_2_ (Supplementary Video 2). **c**, Time-dependent cross-section profiles along the white dashed arrows in **b**, with the distance rescaled to the maximum intensity of the front. **d**, Scheme of a DNA condensate before (top) and after (bottom) invasion. Size increase is not shown for clarity. **e**, CLSM images showing FRAP experiments on DNA condensates before and after invasion. **f**, Recovery profiles of FRAP circles in **e** before and after invasion at 50-mM MgCl_2_. Unless specified, the curves represent the mean values and the shaded areas indicate the s.d. Sample size (*N*) = 4 condensates measured for each condition. The black curve after the axis break shows a 6,000-s-long FRAP experiment on a non-invaded condensate at 20-mM MgCl_2_, proving its arrested state. **g**, Reciprocal of the half-recovery time (1/*t*_1/2_) extracted from **f**. 1/6,000 s^−1^ is taken as 1/*t*_1/2_ for the arrested condensates due to the absence of recovery in FRAP within 6,000 s (mean ± s.d., *N* = 4 condensates measured for both conditions). **h**, Scheme of a fluorescent DNA condensate (p(A_20_-m_Atto488_)_*n*_; magenta) during the invasion of non-fluorescent invaders m*. **i**, CLSM and FLIM images during invasion by 3-μM m* at 50-mM MgCl_2_, with the corresponding phasor plot depicting the lifetime distribution. **j**, Fluorescence lifetime of in-chain fluorophores in arrested/non-invaded and dynamic/invaded regions (mean ± s.d., *N* = 8 condensates). **k**, Scheme of the AFM indentation measurement of the condensates before and after invasion. **l**, Representative force spectroscopy for DNA condensates before and after invasion show distinct force–distance relationships and hysteresis behaviour during compression and retraction of the AFM cantilever. Five independent experiments were performed on the arrested and dynamic condensates (Supplementary Fig. 1). **m**, Dissipation energy for condensates in different states, measured by quantifying the hysteresis area in the compression and retraction curves (mean ± s.d., *N* = 5 condensates measured for each condition). One-way analysis of variance test was used for calculating the *P* values for **g**, **j** and **m**. Scale bars, 10 μm. Source data

To gain mechanistic insights, we synthesized fluorescent p(A_20_-m_dye_)_*n*_ to study the dynamic properties of p(A_20_-m_dye_)_*n*_ chains in the condensate before and after invasion. During invasion with m*-Atto488, the dilution of the p(A_20_-m_Atto594_)_*n*_ condensate in the outer region is now evident (Fig. 2a,b, magenta channel). The approximate fourfold swelling causes a fourfold drop in the intensity of the core material p(A_20_-m_Atto594_)_*n*_. The invader front localizes precisely at the boundary from the compact non-invaded to the swollen invaded condensate (Fig. 2c). We correlate the relative changes in the dynamic properties of the DNA condensates before and after invasion using fluorescence recovery after photobleaching (FRAP) and fluorescence lifetime imaging (FLIM)^39^, which provide complementary insights into the mesoscale diffusion behaviour and nanoscale chain flexibility. FRAP shows drastic differences in recovery kinetics before and after invasion. Before invasion, the condensates are in an arrested state in which the bleached region does not recover even after 6,000 s (Fig. 2d–f). By contrast, after invasion, the condensates become dynamic with fluorescence recovery (Fig. 2e,f). Quantification using the reciprocal of the half-recovery time (1/*t*_1/2_) reveals differences of at least two orders of magnitude (Fig. 2g). FLIM and lifetime analyses show a significant increase from a lifetime of about 2.9 ns in the arrested/non-invaded region to about 3.4 ns in the dynamic/invaded region. The phasor plot shows two different distributions (Fig. 2h–j)^40,41^.

Since our condensates are based on semidilute and entangled ssDNA polymer solutions that are viscoelastic, we used force spectroscopy via colloidal probe atomic force microscopy (AFM)^42^ to study differences in mechanical properties for the condensates before and after invasion (Fig. 2k,l and Supplementary Fig. 1). The force response during indentation increases more drastically for condensates before invasion, whereas after invasion, the condensates are significantly softer. This is a direct comparative measure for the elastic response. Additionally, a larger hysteresis is visible between the compression and retraction curves for condensates after invasion (Fig. 2m and Supplementary Fig. 1). Since hysteresis is a measure of energy dissipation, this indicates increasingly liquid-like properties for condensates after invasion. By contrast, the near-superimposing compression/retraction curves of the condensate before invasion are closer to an elastic material with little energy dissipation. These insights provide direct evidence for distinct mechanical properties of the two types of condensate, as regulated by ballistic wave diffusion.

**Extended Data Fig. 4:**
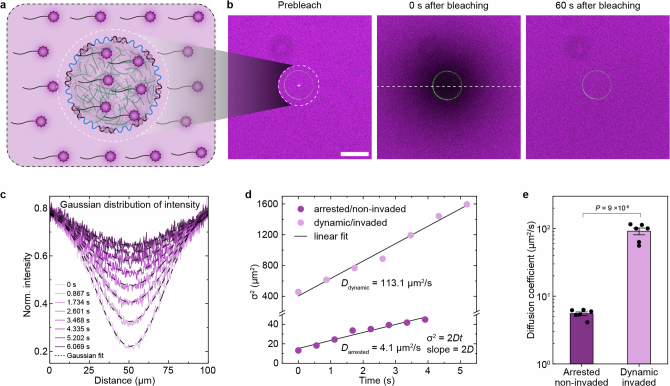
Determination of the diffusion coefficients of short non-binding dummy ssDNA in arrested/non-invaded and dynamic/invaded DNA condensates. **a**, Scheme of a dynamic/invaded DNA condensate immersed in a solution containing Cy5-20nt-dummy. **b**, Representative CLSM images showing point bleaching method on freely diffusing Cy5-20nt-dummy inside a dynamic/invaded non-fluorescent p(A_20_-m)_n_ condensate. The condensates were invaded by 1 μM non-fluorescent m* to become dynamic and mixed with 10 μM Cy5-20nt-dummy (magenta channel). The shell of the condensates was labeled with a small amount of k*-Atto488 (0.5 μM, green channel) for identification, as inside the white dashed circle. 3 s bleaching was applied in the centre pixel (indicated by the white cross in prebleach image) in the condensate before taking post-bleaching images. **c**, Representative cross-sectional line profiles along the white dashed lines in post-bleaching images in **b** for a dynamic/invaded condensate over time, giving Gaussian distribution of the fluorescence intensity, which represents Brownian motion of the bleached Cy5-20nt-dummy strands. The line profiles can be fitted with Gaussian fit to obtain their squared standard deviation (σ^2^) at different time points. σ^2^ changes over time during fluorescence recovery. The diffusion coefficients (*D*) of Cy5-20nt-dummy strands can be extracted by plotting σ^2^ over time with the slope being 2*D*^63^. The same method was also applied on the arrested/non-invaded DNA condensates. **d**, Representative σ^2^ for Cy5-20nt-dummy in arrested/non-invaded and dynamic/invaded condensates, respectively. **e**, Comparison of diffusion coefficients of dummy strands in non-invaded and invaded DNA condensates (mean ± s.d., *N* = 6 condensates measured). One-way analysis of variance test was used for calculating *P* values for **e**. Scale bar is 20 μm. Source data

**Extended Data Fig. 5:**
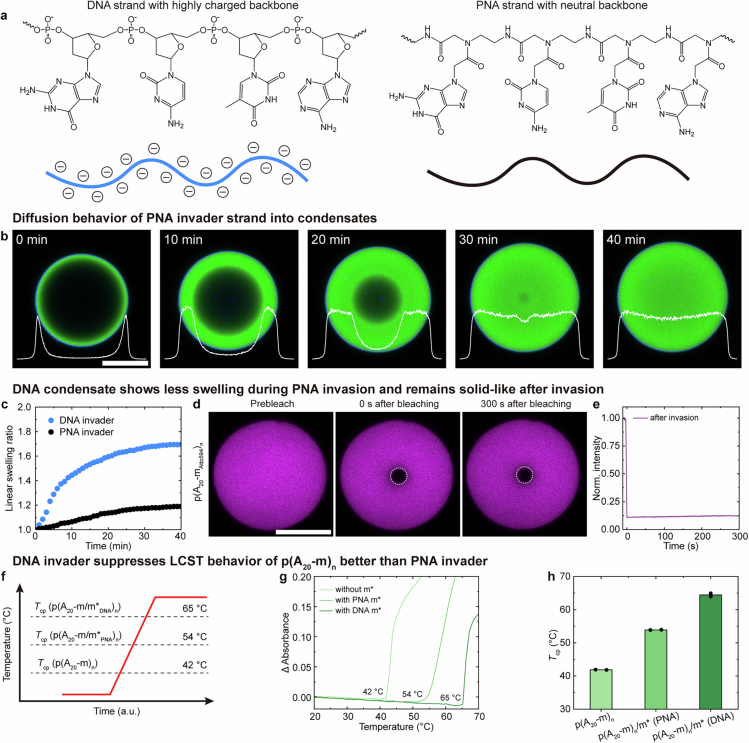
Diffusion mechanism of non-charged PNA invader into DNA condensate. **a**, Structural comparison between a DNA strand and a PNA strand: A DNA strand has a phosphodiester backbone containing negative charges, while a PNA strand is composed of N-(2-aminoethyl)glycine backbone, which is non-charged. PNA binds efficiently to complementary DNA. **b**, Time series CLSM images of DNA condensate (m barcode concentration at 0.4 µM in total solution) during invasion by 5 μM m*_PNA_-Atto488 PNA invader at 50 mM MgCl_2_ (shell labeled with 0.25 μM k*-Atto647, blue channel). **c**, PNA only causes minor swelling during invasion. The swelling kinetics for the DNA invader is the same data as Fig. 1e in main text. **d**, Representative CLSM images showing FRAP experiment on DNA condensates after invasion by m*_PNA_-Atto488 at 50 mM MgCl_2_. **e**, Corresponding intensity recovery kinetics of FRAP circles in **d** showing the arrested nature of the DNA condensates after invasion by m*_PNA_-Atto488 (mean ± s.d., *N* = 4 condensates measured). **f**, Scheme showing the increasing *T*_cp_s when p(A_20_-m)_n_ is hybridized with PNA and DNA m* invaders. **g**, Temperature-dependent change of absorbance at 350 nm of p(A_20_-m)_n_ with and without the existence of PNA or DNA m* invaders. The onset of the increase in absorbance indicates the *T*_cp_. **h**, Comparison of *T*_cp_ for p(A_20_-m)_n_ with and without hybridized PNA or DNA m* invaders (mean, *N* = 2 condensates measured). Scale bars for all are 10 μm. **Summary:** These experiments demonstrate the importance of introducing repulsive charges for regulating the dynamic properties of the invaded condensate. The invasion by a DNA invader brings more charges into the condensate, which generates stronger repulsive electrostatic force within and between DNA chains. Thus, it leads to higher swelling of the invaded condensate and a transition from arrested to dynamic interior. PNA is a poor solubilizer. The absence of a high intensity front in **b** is due to the persistence of the arrested nature of invaded condensates and a lack of swelling in the process. Source data

On the basis of these data, the following picture emerges. The initial DNA condensates are in an arrested state, similar to some aged condensates in biology literature^5,17,19,20,43^. This arrested state is due to the efficient arrest of p(A_20_-m)_*n*_ with divalent Mg^2+^ ions^44^. During invasion, m/m* hybridization occurs by molecular recognition, and invasion is restricted to the frontier zone. The high intensity of the front is due to the initial high concentration of m barcodes in the compact condensate. The m/m* binding causes swelling, dilution of p(A_20_-m/m*)_*n*_, and simultaneous liquefaction in this volume. The relaxation from the compact high-concentration front to the swollen and dynamic final state is a time-dependent process due to the polymeric nature of the condensate. The swelling is induced by chain stiffening and expansion due to partial dsDNA formation in partly hybridized p(A_20_-m/m*)_*n*_, a solubilizing effect of m* binding, and a minor increase in osmotic pressure. Simulations in Extended Data Fig. 3 confirm the expansion, and further control experiments support this physical picture. (1) A non-binding ssDNA diffuses about two orders of magnitude faster in dynamic/invaded condensates than in arrested/non-invaded condensates (Extended Data Fig. 4). The diffusion of small ssDNA is strongly suppressed in the arrested/non-invaded condensates. The measured diffusion coefficients are in line with those found in other condensates^45,46^. (2) Invasion with a non-charged peptide nucleic acid (PNA) strand (m*_PNA_-Atto488) results in substantially less swelling, and the condensates remain in an arrested state after invasion (Extended Data Fig. 5a–e). (3) Hybridization of p(A_20_-m)_*n*_ with DNA m* increases the solubility of p(A_20_-m)_*n*_, whereas PNA-m* has an intermediate effect (Extended Data Fig. 5f–h). Points (2) and (3) confirm that the negative charges of the DNA invader contribute to the swelling and regulation of the arrested-to-dynamic transition of the invaded condensates.

## Reaction–diffusion model and simulations

**Extended Data Fig. 6:**
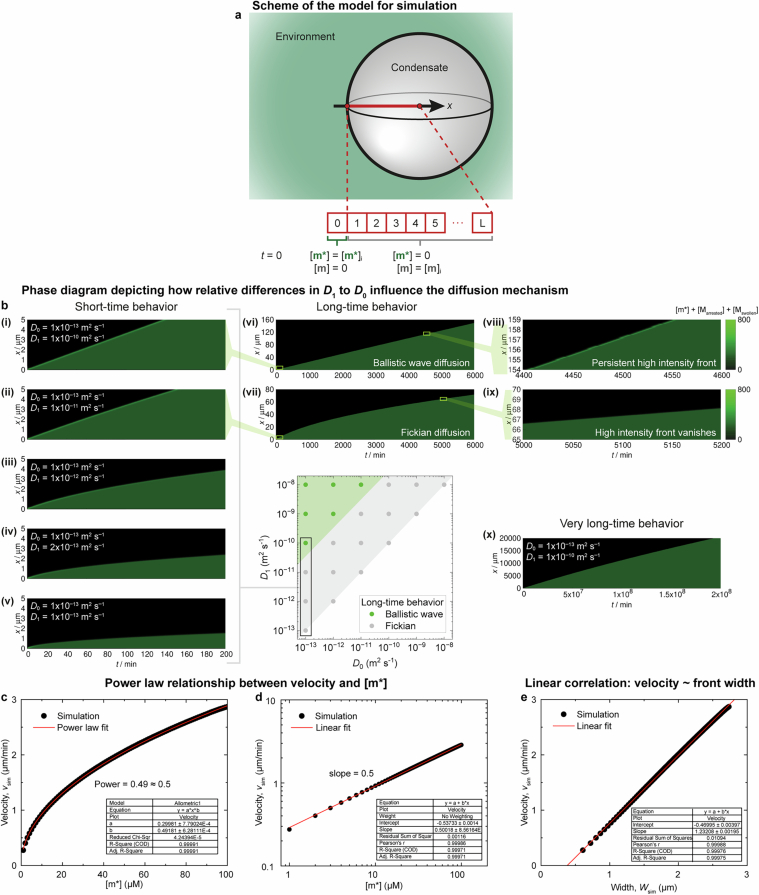
Reaction-diffusion model for simulation of ballistic wave diffusion. **a**, Schematic representation of the one-dimensional mesh used in the numerical model and its relation to the experiments. Initially (*t* = 0), m* is only outside and m is only in the condensate (with a homogeneous distribution). The simulations consider only the radial direction between the surface and the centre of the condensate. **b**, Parameter sweeps for different *D*_1_ and *D*_0_ at constant *k*_bind_ and *k*_swell_ for establishing a phase diagram of different diffusion types. **Key information:** A two order of magnitude difference in *D*_1_ and *D*_0_ is needed for long term ballistic wave diffusion (green area in phase diagram). (See Supplementary Discussion 1 for details); **c**, Simulated velocity (*v*_sim_) of ballistic wave diffusion as a function of invader concentration ([m*]), ranging from 0 to 100 μM. The fitting clearly shows a power law relationship between *v*_sim_ and [m*] with the exponent ≈ 0.5. **d**, The data in **c** plotted in log-log scale with linear fit yielding a slope = 0.5. **e**, *v*_sim_ plotted against simulated width (*W*_sim_), showing a linear correlation in the range of [m*] = 1–100 μM. The experimentally accessible regime is from 0 to 5 µM m*, whereas the simulation enables larger parameter space for probing the power law relationship between front velocity and [m*], and linear correlation between front velocity and front width. Simulated data with [m*] ranging from 0 to 10 μM with smaller step size are plotted in Fig. 4j,l. Supplementary Table 2 lists parameter sets. Source data

To rationalize and predict the behaviour of ballistic wave diffusion, we built a model using reaction–diffusion equations (Extended Data Fig. 6a and Supplementary Methods 2). The model includes different diffusivities of the invader (m*) in the arrested/non-invaded (*D*_0_) and dynamic/invaded part (*D*_1_), the binding reaction of m/m* (*k*_bind_) and the subsequent swelling (*k*_swell_). The swelling is associated with a swelling factor (*f*_swell_). Since we know that volumetric swelling is about fourfold and the m barcode concentration ([m]) inside the condensates is about 1 mM (Supplementary Fig. 2), we can assign realistic starting points to these simulations (Supplementary Table 1).

**Fig. 3:**
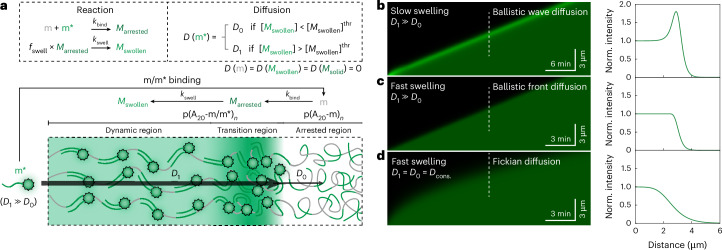
Modelling of ballistic wave diffusion using a reaction–diffusion model. **a**, Chemical reactions and diffusion rules in the ballistic wave diffusion model with a graphical explanation of the processes. **b**–**d**, Representative simulated kymographs (space–time plots) demonstrating ballistic wave diffusion (**b**), ballistic front diffusion (**c**) and Fickian diffusion (**d**) at different conditions. The right plots show individual front shapes by cross-sectional line profiles taken along the white dashed lines in the corresponding kymographs. Supplementary Table 2 lists the parameter sets. Source data

Let us hypothesize a ballistic wave diffusion process. When m* diffuses into the condensate, it binds to an unoccupied m and forms a compact p(A_20_-m/m*)_*n*_ that is part of the arrested interior (m/m* = *M*_arrested_). After binding, solubilization of the local segment occurs, leading to swelling and a transition from *M*_arrested_ to *M*_swollen_. Binding can be considered diffusion-controlled and unbinding can be neglected because of the high binding strength of m/m* (melting temperature, *T*_m_ ≈ 71.5 °C; Supplementary Methods 3). Once swelling has occurred, that is, [*M*_swollen_] exceeds a threshold, *D*_0_ for m* in this volume element increases to *D*_1_ (*D*_1_ ≫ *D*_0_). Therefore, subsequent m* can diffuse rapidly through the swollen region, but is drastically slowed down at the transition to the arrested region (Fig. 3a).

This model can successfully reproduce ballistic wave diffusion with ballistic propagation kinetics (Δ*X* ∝ *t*) and a sharp and stably propagating diffusion front of high intensity with a locally high m/m* concentration (Fig. 3b). It can also extrapolate behaviour. For instance, when the swelling process is accelerated (*k*_swell_ increases) but the difference between two diffusion coefficients is maintained, the high-intensity front (that is, the wave) disappears, but the front remains sharp and still propagates linearly with time (Fig. 3c). We term such a state as ‘ballistic front diffusion’. When the diffusion coefficient for m* is assumed to be the same inside the arrested/non-invaded and dynamic/invaded parts of the condensate, the transport follows Fickian diffusion with a blurry front and a front displacement proportional to *t*^1/2^ (Fig. 3d). A full phase diagram with varied *D*_0_ and *D*_1_ shows that the appearance of the ballistic front and wave diffusion originates from the difference between *D*_0_ and *D*_1_ (Extended Data Fig. 6b and Supplementary Discussion 1). Additional orienting simulations in two and three dimensions are in line with the one-dimensional simulations and show a slight acceleration at late stages, as observed in experiments (Fig. 1c,d, Supplementary Fig. 3 and Supplementary Methods 4).

**Fig. 4:**
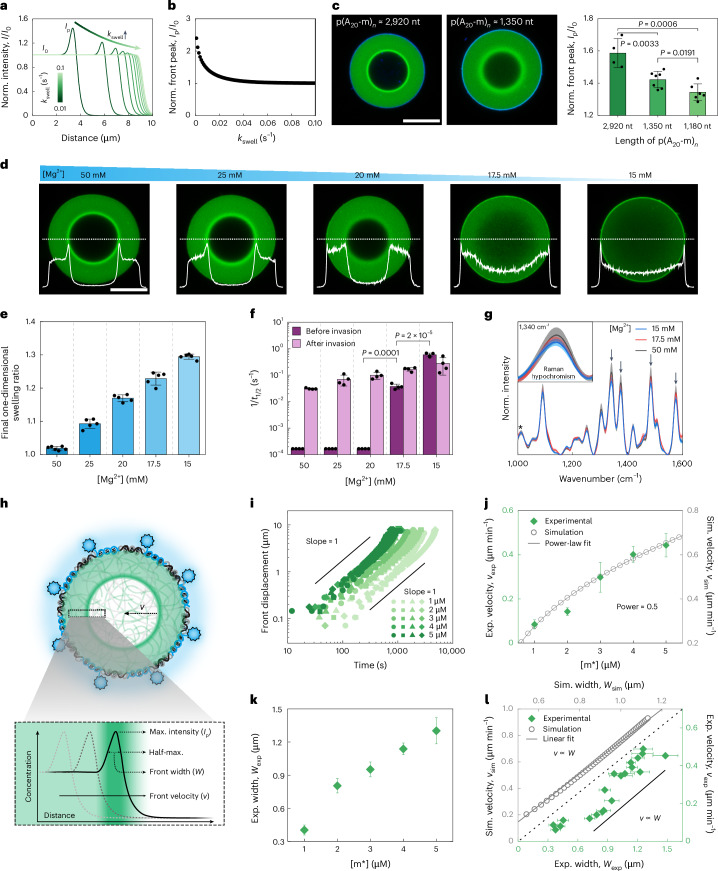
Understanding and tuning the diffusion mechanism and kinetics by combined simulations and experiments. **a**, Simulated intensity profiles (normalized by intensity at a fully swollen region, *I*_0_) of ballistic wave diffusion show a decrease in the front height for enhanced swelling kinetics (*k*_swell_ increases). **b**, Simulated peak intensity (*I*_p_) normalized by *I*_0_ as a function of *k*_swell_. **c**, CLSM images and quantification show a reduced front peak for shorter p(A_20_-m)_*n*_ chains (total length in nucleotides (nt); mean ± s.d., *N* = 4, 7 and 6 condensates measured for 2,920, 1,350 and 1,180 nt). **d**, CLSM shows a transition from ballistic wave to Fickian diffusion when reducing [Mg^2+^]. The white lines depict the cross-sectional intensity profiles. Experiments were repeated at least two times for each [Mg^2+^] with similar results. **e**, [Mg^2+^]-dependent swelling (one-dimensional) of non-invaded condensates (mean ± s.d., *N* = 6 condensates measured for 50-mM Mg^2+^, and *N* = 5 condensates measured for all other conditions). **f**, [Mg^2+^]-dependent 1/*t*_1/2_ of DNA condensates from FRAP experiments before and after invasion (mean ± s.d., *N* = 4 condensates measured for all the conditions). **g**, [Mg^2+^]-dependent Raman spectroscopy of DNA condensates before invasion. Spectra are normalized to the peak at 1,014 cm^–1^ of the deoxyribose (*) (mean ± standard error, *N* = 10, 9 and 8 condensates measured for 50, 17.5 and 15 mM of Mg^2+^). The black arrows indicate Raman hypochromism, with one magnification around 1,340 cm^–1^. **h**, Scheme for the quantification of front velocity (*v*) and front width (*W*) of ballistic wave diffusion. **i**, Representative time-dependent front displacement during ballistic wave diffusion at various invader concentrations. **j**, *v* as a function of invader concentration (mean ± s.d., *N* = 4 condensates measured for all the conditions). **k**, *W* as a function of invader concentration (mean ± s.d., *N* = 4 condensates measured for all the conditions). Here the mean of *W* is calculated for each invader concentration. **l**, *v* plotted against *W* shows the linear correlation (mean ± s.d. of *W* in each experiment; *W* is measured six times for each invasion process at different times). The axis scales for the simulated and experimental results are scaled but show similar relationships. Supplementary Table 2 lists the parameter sets. One-way analysis of variance test was used for calculating the *P* values for **c** and **f**. Scale bars, 10 μm. Source data

**Extended Data Fig. 7:**
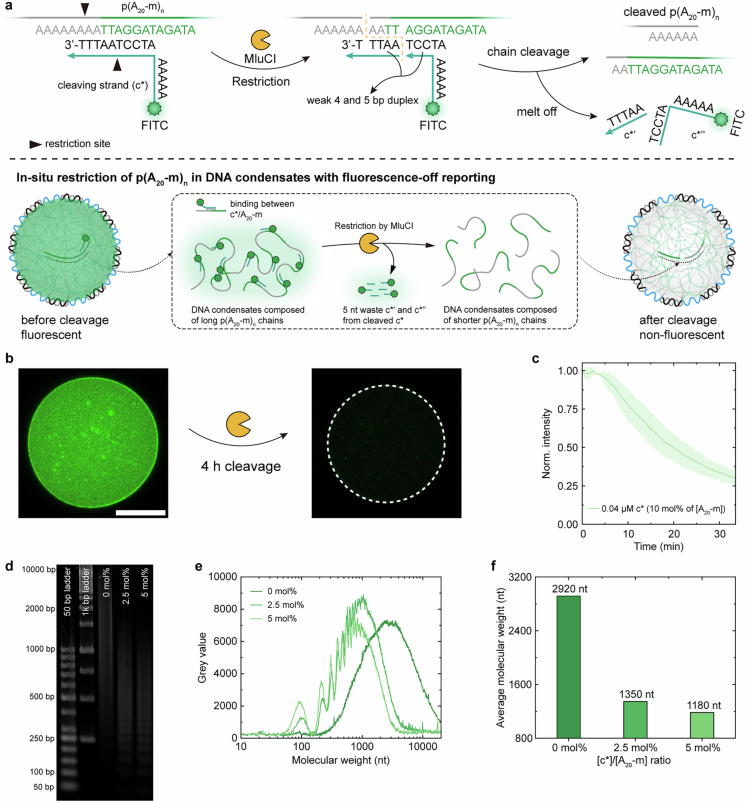
Preparation of DNA condensates containing shorter p(A_20_-m)_n_ chains by in-condensate restriction. **Rationale:** Shortening of the p(A_20_-m)_n_ strands cannot be done before condensate preparation because excessively shortened strands might not integrate into the condensates due to the molecular weight dependence of the LCST behavior^31^. Hence, we applied restriction inside the condensate. **a**, Scheme for the design of the enzymatic restriction by MluCI on p(A_20_-m)_n_ chains with help of a cleaving strand (c*) inside the condensates. c* hybridizes to p(A_20_-m)_n_ and generates a recognition site for the restriction enzyme MluCI. The ratio of [c*]/[A_20_-m] codes the extent of cleavage. c* contains a spacer (A_5_) with a fluorophore (FITC) at the 5’ end. Before cleavage, c* has 10 base pairs with p(A_20_-m)_n_ (*T*_m_ ≈ 40.2 – 44.4 °C, Supplementary Method 3) and will thus bind tightly at experimental temperature (room temperature). After cleavage, both subparts (c*’ and c*”) of the c* only have 5 base pairs with p(A_20_-m)_n_ (*T*_m_ ≈ 0 °C and 3.4 – 11.6 °C, respectively, Supplementary Method 3) and will thus melt off. The fluorophore at the 5’ end of the cleaving strand serves as a reporter for monitoring the restriction as fluorescence vanishes from the condensates into the surrounding. The finally cleaved DNA condensates will not contain any cleaved subparts of c* due to their low *T*_m_s, but only contain shorter p(A_20_-m)_n_ chains. **b**, Representative CLSM images showing the enzymatic restriction of DNA condensates loaded with sub-stoichiometric amount c* (10 mol%). **c**, Restriction kinetics (light green) indicated by decrease of normalized fluorescence intensity within DNA condensate. Conditions: 0.04 μM c* = 10 mol% of [A_20_-m], 0.5 U/μL MluCI enzyme, 15 mM MgCl_2_, room temperature (mean ± s.d., *N* = 2 kinetic experiments). **d**, Gel electrophoresis of non-cleaved (with 0 mol% c*) and cleaved p(A_20_-m)_n_ (with 2.5 and 5 mol% c*). The gel electrophoresis image is background-subtracted. **e**, Quantification of molecular weight distribution by grey value profiles extracted from **d**. 50 bp and 1k bp ladders are used for the calibration of molecular weight as a function of migration distance. The molecular weight is reported as nucleotide number (nt), corresponding to double of the calibrated base pair number (bp). **f**, Number average molecular weight of individual p(A_20_-m)_n_ chains calculated from the molecular weight distributions of individual lanes in **e**. Gel source data is provided. Scale bar is 10 μm. Source data

Simulations suggest that the sharpness and height of the front (*I*_p_/*I*_0_) should decrease with enhanced swelling kinetics (Fig. 4a,b). At the molecular level, swelling kinetics depend on the length of the p(A_20_-m)_*n*_ chains because swelling occurs through relaxation, reptation and disentanglement of the polymer chains^47^. Indeed, we find a less-pronounced high-intensity wave for shorter p(A_20_-m)_*n*_ (Fig. 4c and Extended Data Fig. 7).

**Extended Data Fig. 8:**
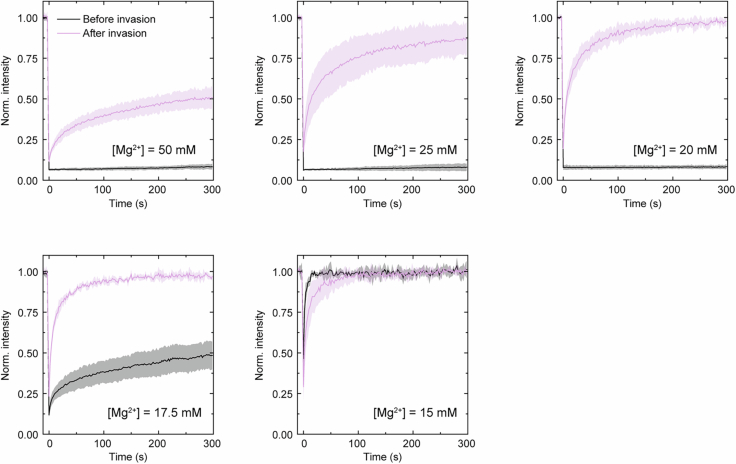
FRAP of DNA condensates before and after invasion at different [Mg^2+^]. FRAP experiments performed on DNA condensates of p(A_20_-m_Atto594_)_n_ before (black) and after (violet) invasion by 1 μM m*-Atto488 at the MgCl_2_ concentration mentioned inside the panels (mean ± s.d., *N* = 4 condensates measured for all conditions). The plots shown here are used for the determination of 1/*t*_1/2_ for Fig. 4f in the main text. Source data

Moreover, the simulations predict a transition from ballistic wave diffusion to ballistic front diffusion and even to classical Fickian diffusion for condensates with increasing swelling kinetics and when the differences in *D*_1_ and *D*_0_ of m* are reduced (Fig. 3b–d). This can be experimentally achieved by decreasing the ionic strength. Lower [Mg^2+^] increases the net charge density of the phosphodiester backbone of p(A_20_-m)_*n*_ (refs. ^48,49^). In addition to increasing the persistence length of p(A_20_-m)_*n*_, the chain-bridging effect of Mg^2+^ is also reduced^44^. This is reflected in a Mg^2+^-dependent swelling of DNA condensates (Fig. 4e). A gradual decrease in [Mg^2+^] from 50 mM to 15 mM leads to a change in the invasion process from ballistic wave diffusion to ballistic front diffusion and finally to Fickian diffusion (Fig. 4d and Supplementary Video 3). FRAP experiments on non-invaded and invaded DNA condensates allow the quantification of internal dynamics (Fig. 4f and Extended Data Fig. 8). For [Mg^2+^] ≥ 20 mM, the condensates are arrested before invasion and show a dynamic character only after invasion. At this point, the swelling due to lower [Mg^2+^] before invasion does not yet overcompensate for the interaction between the p(A_20_-m)_*n*_ chains. Further decrease in [Mg^2+^] yields a discontinuous and drastic property change. At the transition point to Fickian behaviour ([Mg^2+^] = 17.5 mM), the DNA condensates already exhibit a comparatively dynamic interior that still increases by one order of magnitude after invasion. Lower [Mg^2+^] (15 mM) then shows similar dynamics before and after invasion. This clearly underscores two points. First, the observation of sharp diffusion fronts requires a distinct transition from an arrested to a dynamic condensate during invasion. Second, the transition between the two regimes (and thus the appearance of a high-intensity wave) depends on preinvasion swelling, because this eliminates the need for a swelling process after the invader binds to its barcode.

Broadband coherent anti-Stokes Raman scattering (BCARS) provides additional information on the chain conformation of p(A_20_-m)_*n*_. Lower [Mg^2+^] leads to a marked decrease in Raman intensity for specific peaks in the range of 1,300–1,600 cm^–1^ (Fig. 4g), characteristic of nucleobases^50^. This phenomenon, called Raman hypochromism, suggests that the p(A_20_-m)_*n*_ chains in the condensates adopt a more compact coil conformation with less base stacking due to geometric confinement at high [Mg^2+^]. By contrast, lower [Mg^2+^] leads to a more extended, relaxed conformation due to an increase in the effective charges along the backbone, resulting in the swelling of the condensates and a gain in free volume and dynamics, which facilitates the base stacking interactions known for adenine in ssDNA^51–53^. Using a fluorescent reporter for Mg^2+^, we find a slightly higher Mg^2+^ concentration inside the condensates than the solution before invasion, whereas such differences vanish after the invasion process. This arises from the lower negative charge density inside the condensates after invader-induced swelling (Supplementary Fig. 4).

Finally, we ask how well the model can capture and predict the tunability of the frontal velocity and the width of the high-intensity diffusion front. Our approach targets establishing generic scaling laws rather than a perfect fit that would require accurate assumptions for unknown kinetic parameters. Simulations predict a power-law dependence of the front velocity (*v*_sim_) on the concentration of the invader (*v*_sim_ ∝ [m*]^0.5^; Fig. 4j and Extended Data Fig. 6c,d). This dependence is reflected in experiments (Fig. 4i,j). A similar good agreement is found for the dependence of the front velocity (*v*) with front width (*W*), which follows a linear correlation (*v* ∝ *W*; Fig. 4h,k,l and Extended Data Fig. 6e). The agreement between the model and experiment provides a clear physical picture of the dynamization and relaxation processes at the diffusion front. Higher [m*] can penetrate deeper into the arrested region at the steady-state diffusion front, resulting in a wider front interface and transition region. Accordingly, more compact p(A_20_-m)_*n*_ chains bind m* in a given volume and time element, leading to faster relaxation in a larger volume element and, consequently, to a faster transition from compact p(A_20_-m)_*n*_ to swollen p(A_20_-m/m*)_*n*_.

## Conclusions

**Extended Data Fig. 9:**
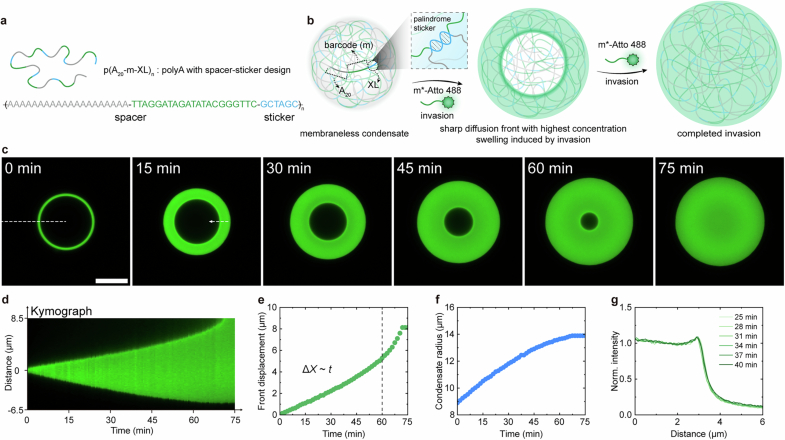
Ballistic wave diffusion in membraneless DNA condensates with spacer-sticker model. **a**, p(A_20_-m-XL)_n_ sequence design of adenine-rich DNA polymer with palindromic domain (XL) mimicking spacer-sticker model. XL domains are self-complementary DNA sequences that serve as crosslinks. **b**, Schematic representation of the ballistic wave diffusion occurring during the invasion of m*-Atto488 into such membraneless p(A_20_-m-XL)_n_ DNA condensates. **c**, Time-series CLSM images showing the invasion process of invaders (m*-Atto488, 3 μM; green; 50 mM MgCl_2_). **d**, Kymograph of the invasion process along the white dashed line in **c**. **e**, Front displacement during invasion is linear with time. Acceleration happens at the end stage (noted with the dashed line) because of the interference between diffusion fronts from all radial directions. **f**, Radius of DNA condensate during invasion. **g**, Cross-sectional line profiles taken at various times along the white dashed arrow in **c**. The distances are rescaled to the maximum intensity of the wave to highlight the self-similar shape during invasion. See also Supplementary Video 4. Scale bar is 10 μm and applies to all micrographs in **c**. Source data

We have identified a new diffusion mechanism—ballistic wave diffusion—in soft matter in general and in biomolecular condensates in particular. Unlike Fickian diffusion, ballistic wave diffusion is characterized by an ultrasharp diffusion front that propagates with ballistic properties, that is, linearly with time. The invading molecules accumulate in a non-equilibrium high-intensity front, representing a transition interface that relaxes with time. Molecular recognition and a transition from arrested to dynamic and swollen condensates during the invasion of a binding macromolecule are critical elements for ballistic wave diffusion. This diffusion mechanism could not have been previously identified in classical polymer coacervates, because the molecular recognition event between the invader and its ‘receptor’ plays a key role in the non-equilibrium process. To verify the general relevance of ballistic wave diffusion, we also investigate the invasion of m* into a membraneless sticker–spacer DNA condensate formed by p(A_20_-m-XL)_*n*_, as internally stabilized by palindromic XL domains. Indeed, ballistic wave diffusion also manifests there (Extended Data Fig. 9). This is an important generalization as such membraneless sticker–spacer condensates are generic models for membraneless organelles formed by RNA or proteins in biology^15,54,55^. In future, it will be relevant to understand how domain variations, such as extending or reducing the adenine repeats, and modulating thermodynamic binding interactions, by introducing mismatches in the invader sequence or changing its length, influence the diffusion behaviour.

**Extended Data Fig. 10:**
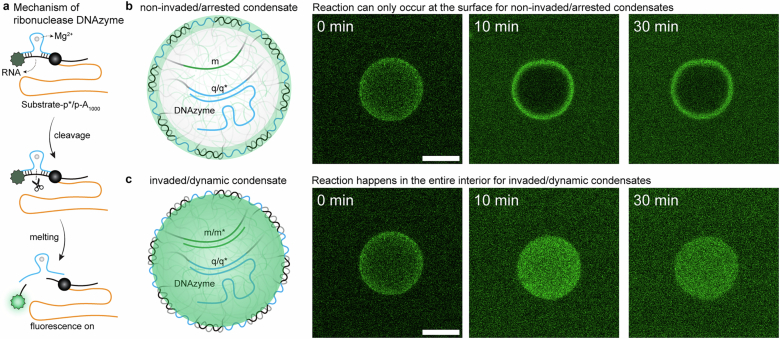
Catalytic cleavage by ribonuclease DNAzyme in DNA condensates is regulated by different dynamic states. **a**, Schematic representation for the catalytic cleavage process of p*-Substrate/p-A_1000_ by 10-23 ribonuclease DNAzyme tethered via additional q/q* domains. The DNAzyme is able to break RNA bonds once the substrate is hybridized with the blue recognition parts. DNA condensates containing 80 % m barcode and 20 % q barcode (obtained by using a mixture of p(A_20_-m)_n_ and p(A_20_-q)_n_ during condensate formation) were used for DNAzyme encapsulation by q/q* hybridization. **b**, Before invasion, DNA condensates are in arrested state. The catalytic cleavage of p*-Substrate at the DNAzyme encapsulated (by q/q* hybridization) in the arrested condensates only happens at the condensate surface. **c**, After invasion with m*, the DNA condensates are in a dynamic state. Now the catalytic cleavage of the DNAzyme can happen in the entirety of the condensate. The m* invader has served as a gate opener for the catalysis. Scale bars are 10 μm.

Although we show ballistic wave diffusion in purely synthetic DNA model condensates, molecular recognition (for example, based on protein/RNA binding or coiled-coil interactions) and different dynamic states are ubiquitous in biological coacervates^2,15,17,20,24,28^. Interestingly, sharp diffusion fronts can be found in biology literature, for example, for condensates based on coiled-coil interactions^28^, but without noticing its importance or physical origin. In particular, our study explains these observations with non-Fickian diffusion concepts, bridging the gap between polymer physics and cellular processes. Although the implications of this observation for biological function and its generalization remain to be elucidated, the importance of ballistic wave diffusion lies in its ability to generate sharper signal fronts with clearer spatial concentration thresholds compared with traditional Fickian diffusion. Moreover, the ballistic wave diffusion mechanism highlights how invading molecules can—in real time—modify the dynamic properties in condensates. This has important implications for the spatial and temporal sequestration of molecules within cellular condensates and for controlling transport processes at membranes, such as the nuclear pore complex^7^. Besides, it suggests an additional role of condensate dynamics with respect to signal selection and rejection, and for how biology might adaptively regulate these processes through environmental triggers that alter the condensate dynamics. As a proof of concept, we encapsulate a ribonuclease^56^ inside our condensates and find that the arrested condensates before invasion reject the substrate from diffusing into the condensate, and the reaction only occurs at the condensate surface. By contrast, dynamic condensates after invasion enable reactions in the entire interior (Extended Data Fig. 10). This demonstrates a functional gating effect related to the condensate dynamics as regulated by ballistic wave diffusion.

Our study suggests that ballistic wave diffusion can be a potentially important pathway for cells to regulate the dynamics of condensates and prevent them from aging and aggregation, which guarantees their normal functions during cellular processes. This could potentially guide therapeutic solutions for neurodegenerative diseases, which typically involve the liquid–solid transition of relevant biomolecular condensates formed by various proteins and nucleic acids, such as hnRNPA1, TDP-43 and FUS^19,57–59^. One may speculate that because fibre formation was observed to occur at the surface of such condensates^60^, their frontal suppression could provide an effective therapeutic intervention. We anticipate that our study provides a new perspective to comprehend the intricate interplay of dynamics and chemical kinetics in biomolecular condensates and their relation to cellular function.

## Methods

### Materials

ssDNA was purchased from Biomers and Integrated DNA Technologies. PNA was purchased from PANAGENE. Supplementary Data 2 summarizes all the sequences. T4 DNA ligase (2 U μl^–1^), exonuclease I (40 U μl^–1^), exonuclease III (200 U μl^–1^) and Φ_29_ polymerase (10 U μl^–1^) were purchased from Lucigen. Thermostable inorganic pyrophosphatase (2 U μl^–1^), inorganic pyrophosphatase (0.1 U μl^–1^), MluCI (10 U μl^–1^), terminal transferase (20 U ml^–1^) and nuclease-free water were bought from New England BioLabs. Deoxynucleotide triphosphate (dATP, dTTP, dGTP and dCTP) (100 mM), aminoallyl-dUTP-XX-ATTO-488 (1 mM) and aminoallyl-dUTP-XX-ATTO-594 (1 mM) were purchased from Jena Bioscience. Hexadecane, NaCl, MgCl_2_, Tris(hydroxymethyl)-aminomethane hydrochloride (Tris-HCl, pH 8), Trizma base, acetic acid and ethylenediaminetetraacetic acid disodium salt dihydrate (EDTA) were purchased (as bioreagent grade, if available) from Sigma-Aldrich. Agarose Low EEO was purchased from AppliChem. RNase inhibitor (40 U μl^–1^), RNase-free TE buffer (Invitrogen, 10-mM Tris and 1-mM EDTA, pH 8.0, 500 ml), SYBR Gold, GeneRuler 50-bp DNA ladder, GeneRuler 1-kb DNA ladder, Mag-fluo-4 AM and Duke Standards dry borosilicate glass microspheres (18.2 ± 1 μm) were purchased from Thermo Fisher Scientific. 384-well high-content imaging glass-bottom microplates were purchased from Corning. AFM probe cantilevers (HQ:NSC36/tipless/No Al) with three different AFM cantilevers having spring constants between 0.6 N m^–1^ and 2.0 N m^–1^ were purchased from MikroMasch.

### Instruments

Thermal heating ramps were performed on a TPersonal Thermocycler (Analytik Jena). Incubation with shaking was carried out on an Eppendorf ThermoMixer C. DNA concentrations were determined by a ScanDrop (Analytik Jena) and DS-11 spectrophotometer (DeNovix). Confocal laser scanning microscopy (CLSM) was performed on a Leica Stellaris 5 device. FLIM was performed on a Leica Stellaris 8. Fluorescence microscopy experiments for monitoring the swelling kinetics of DNA condensates after diluting to lower [Mg^2+^] were performed on EVOS M7000 (Invitrogen). Temperature-dependent ultraviolet–visible measurements were performed on ScanDrop (Analytik Jena) connected with a temperature controller (JUMO dTRON 308). Gel electrophoresis was performed in a gel chamber from Biostep. Fluorescence measurements were performed using a plate reader (Tecan). Raman fingerprint measurements were performed by a custom-built BCARS microscope (details below). AFM colloid probes were fabricated by attaching glass beads to the tipless AFM cantilevers using a three-axis oil hydraulic micromanipulator (MMO-203 from Narishige) under a bright-field optical microscope (ZEISS Axiotech). AFM measurements were performed using a JPK SPM NanoWizard III device with a liquid sample cell on top of an inverted microscope stage (ZEISS Axiovert).

### Synthesis of circular ssDNA templates and long ssDNA polymers

The syntheses of the circular DNA template and its corresponding ssDNA polymer are adapted from our previous report with slight modifications (Extended Data Fig. 1)^31,33^. The linear ssDNA template and the corresponding ligation strand were first mixed at a concentration of 1 μM in 100 μl of TE buffer containing 100 mM of NaCl. The solution was heated to 85 °C for 5 min before cooling to 25 °C at a cooling rate of 0.01 °C s^–1^ for complete hybridization. Afterwards, 20 μl of 10× ligase buffer (500 mM of Tris-HCl, 100 mM of MgCl_2_, 50 mM of dithiothreitol and 10 mM of ATP (Lucigen)), 70 μl of nuclease-free water and 10 μl of T4 DNA ligase (2 U μl^–1^ (Lucigen)) were introduced into the reaction mixture and gently mixed before leaving at room temperature for a 3-h reaction. The reaction mixture was then heated to 70 °C for 20 min to deactivate the enzyme. Then, 10 μl of exonuclease I (40 U μl^–1^ (Lucigen)) and 10 μl of exonuclease III (200 U μl^–1^ (Lucigen)) were added into the reaction mixture for further overnight reaction at 37 °C to fully remove the ligation strands and any non-circularized templates in solution. Afterwards, the reaction mixture was heated to 80 °C for 40 min to deactivate the enzymes. To obtain the final circular ssDNA templates, the reaction mixture was washed by adding 400 μl of TE buffer and filtrated using Amicon ultracentrifugal filters with a 10-kDa cut-off (Merck Millipore) three times. The concentrations of the collected circular ssDNA templates were measured by the DS-11 spectrophotometer (DeNovix), and the templates were stored in a TE buffer at –20 °C.

For the synthesis of the long ssDNA polymers, we used rolling circle amplification. Here 5 μl of a circular template (1 μM in a TE buffer) and 1 μl of an exonuclease-resistant primer (10 μM in TE buffer) were mixed with 76 μl of nuclease-free water, 10 μl of commercial 10× polymerase buffer (500 mM of Tris-HCl, 100 mM of (NH_4_)_2_SO_4_, 40 mM of dithiothreitol and 100 mM of MgCl_2_ (Lucigen)), 2 μl of Φ_29_ DNA polymerase (10 U μl^–1^ (Lucigen)), 20 μl of inorganic pyrophosphatase (0.1 U μl^–1^ (New England BioLabs)) and 5 μl of adjusted deoxyribose nucleoside 5′-triphosphate mix (100 mM, the mix contains pure dATP, dTTP, dCTP and dGTP solutions mixed in corresponding proportions of the exact composition of the desired ssDNA polymer repeating units (Jena Bioscience)). Note that for the synthesis of ssDNA polymers with in-chain fluorophores of Atto488 and Atto594, that is, p(A_20_-m_Atto488_)_*n*_, p(T_20_-k_Atto488_)_*n*_ and p(A_20_-m_Atto594_)_*n*_, we replaced 2 mol% of the dTTP in the mix with either aminoallyl-dUTP-XX-ATTO-488 or aminoallyl-dUTP-XX-ATTO-594 for random insertion of the dye along the ssDNA chains during rolling circle amplification. The reaction mixture was kept at 30 °C for 50 h before thermal cleavage at 95 °C for 15 min to shorten the ultrahigh-molecular-weight ssDNA^31^. The final products were purified by rinsing with 400 µl of TE buffer and filtration in Amicon Ultracentrifugal filters (30-kDa cut-off; Merck Millipore) three times. The concentrations of the collected final ssDNA polymers were measured using the DS-11 spectrophotometer (DeNovix), and the DNA polymers (cleaved at 95 °C for 15 min) were stored in a TE buffer at –20 °C.

### Preparation of DNA condensates via liquid–liquid phase separation

The preparation of the DNA condensates is adapted from our previous reports with modifications for the formation of larger DNA condensates (Extended Data Fig. 1)^31,33^. For core–shell condensates, p(A_20_-m)_*n*_ (0.5556 g l^–1^) and p(T_20_-k)_*n*_ (0.0694 g l^–1^) were mixed in a TE buffer without any salt at a final volume of 9 μl. The solution mixture was then heated at 95 °C for 15 min (in addition to the first thermal cleavage during the ssDNA synthesis mentioned above) for thermal cleavage to further reduce the chain length of the ssDNA polymers. Afterwards, 1 μl of TE buffer containing 500 mM of MgCl_2_ was introduced into the reaction mixture. The solution containing finally 0.5 g l^–1^ of p(A_20_-m)_*n*_ and 0.0625 g l^–1^ of p(T_20_-k)_*n*_ with 50 mM of MgCl_2_ were heated to 95 °C for 20 min and subsequently cooled down to room temperature at 3 °C s^–1^ (ref. ^31^). Finally, a 10-μl solution containing the core–shell DNA condensates was diluted five times by adding 40 μl of TE buffer containing various amounts of MgCl_2_ to adjust [Mg^2+^] from 15 mM to 50 mM. The obtained 50-μl DNA condensates solution (as diluted five times) has 0.1 g l^–1^ of p(A_20_-m)_*n*_ and 0.0125 g l^–1^ of p(T_20_-k)_*n*_, corresponding to ~8 μM of m barcode and ~1 μM of k barcode, respectively, in the total solution. The solution was then stored in a fridge at 4 °C for 1 week for equilibration. Before usage, the condensate solutions were always slightly vortexed for redispersion. For the membraneless condensate formed by p(A_20_-m-XL)_*n*_, the same method was used, except p(T_20_-k)_*n*_ was not added in the solution. All the heating ramps were performed in polymerase chain reaction tubes with negligible evaporation. All imaging was done by CLSM at room temperature after preparation.

The chain lengths of p(A_20_-m)_*n*_ and p(T_20_-k)_*n*_ for the DNA condensates were quantified by gel electrophoresis using 2 wt% agarose gel under 5 V cm^–1^ for 150 min in a TAE buffer containing 40 mM of Tris-HCl, 20 mM of acetic acid and 1 mM of EDTA (Extended Data Fig. 1b). Post-staining by SYBR Gold was applied. Quantification of chain length distribution and average chain lengths of p(A_20_-m)_*n*_ and p(T_20_-k)_*n*_ uses a previously reported method^61,62^.

### Ballistic wave diffusion kinetics

Here 19 μl solution containing k*-Atto647 (0–0.5 μM), m*-Atto488 (1–5 μM) and the desired concentration of MgCl_2_ in a TE buffer was prepared and homogeneously mixed before the final addition of 1 μl of DNA condensate stock solution (diluted five times). The DNA condensates were, thus, diluted 100 times, equalling a content of 0.4 µM of m barcode in the solution. Then, 20 μl of the mixture was added into a 384-well plate with another 10–20 μl of hexadecane on top to prevent evaporation. Typically, DNA condensates with diameters of around 15 ± 2 μm were chosen for the observation and recording of the invasion process. The invasion process was followed by taking time-series CLSM images with identical settings. The displacement of the front was calculated in reference to the centre of the condensate. Due to the change in the centre plane of the DNA condensates along the *z* direction during invasion-induced swelling, the *z*-focused cross-sectional plane in the CLSM was always slightly adjusted before each image acquisition to record the diffusion in the same two-dimensional plane.

### Determination of interior dynamics of DNA condensates by FRAP

To determine the dynamic properties of the DNA condensates before and after invasion, we used the region of interest (ROI) bleaching method (Figs. 2d–g and 4f and Extended Data Fig. 8). DNA condensates composed of p(A_20_-m_Atto594_)_*n*_ were first imaged with a low laser intensity five times (2 s per frame) as the prebleaching images, before bleaching three times in a small circular ROI with a diameter of 2 μm by 100% laser intensity in the 561-nm channel. Then, 150 frames of the post-bleaching images were recorded at a rate of 2 s per frame over 300 s. The intensities within the circular ROI (*I*_ROI_) and intensities in a circular region of the same size away from the bleached region within the condensates (*I*_ref_) in the pre- and post-bleaching images were measured with ImageJ (v2.9.0; 64-bit) for performing double normalization in the bleached regions as

1 $${I}_{{\rm{Norm}}}(t)=\frac{{I}_{{\rm{ROI}}}(t)}{{I}_{{\rm{ROI}}}({t}_{0})}\times \frac{{I}_{{\rm{ref}}}({t}_{0})}{{I}_{{\rm{ref}}}(t)}$$

to quantify the recovery kinetics over time. Note that *I*(*t*_0_) represents the intensity measured in the first image before bleaching. The half-recovery times (*t*_1/2_) from individual experiments were extracted from their corresponding recovery kinetics.

Note that the FRAP protocol is different for the 6,000-s FRAP experiment for the confirmation of arrested state of non-invaded DNA condensates at 20-mM MgCl_2_ (Fig. 2f). For this experiment, the post-bleaching images were manually recorded every several minutes over 6,000 s. Before each image acquisition, the focal plane was tuned to the bleached plane of the condensates to make sure the same ROI was monitored, avoiding interference caused by the movement of DNA condensate over the long experimental time. Due to the absence of recovery in this FRAP experiment over 6,000 s, we take 6,000 s as *t*_1/2_ for all the arrested DNA condensates.

### FLIM experiments

To determine lifetime changes in the in-chain fluorophores along the p(A_20_-m)_*n*_ chain during ballistic wave diffusion, we used DNA condensates with p(A_20_-m_Atto488_)_*n*_ chains. We used non-fluorescent m* as the invader to avoid unnecessary interference between dyes. The reaction mixture contained diluted DNA condensates (0.8 μM of m barcode) and 3 μM of m* in a TE buffer with 50 mM of MgCl_2_.

FLIM images were recorded (Fig. 2h–j) based on FALCON-modified time-correlated single-photon-counting method with a Leica Stellaris 8 FLIM module. The phasor plots were generated by transforming the lifetime determined at each pixel of the FLIM image into a two-dimensional map according to the following equations^41^:

2 $${g}_{{\rm{i}},{\rm{j}}}\left(\omega \right)={\int }_{\!0}^{{T}}I(t)\cdot \cos \left(n\omega t\right){\rm{d}}t/{\int }_{\!0}^{{t}}I(t){\rm{d}}t,$$

3 $${s}_{{\rm{i}},{\rm{j}}}\left(\omega \right)={\int }_{\!0}^{{T}}I(t)\cdot \sin \left(n\omega t\right){\rm{d}}t/{\int }_{\!0}^{{t}}I(t){\rm{d}}t,$$

where *g*_*i*,*j*_(*ω*) and *s*_*i*,*j*_(*ω*) are the *x* and *y* coordinates of the phasor plot, respectively; *n* and *ω* are the harmonic frequency and angular frequency of excitation, respectively; and *T* is the repeat frequency of acquisition. Major distributions of phasors were identified by cursors in the phasor plot, and the corresponding lifetimes were assigned into the greyscale images in red and blue colours.

### Determination of diffusion coefficients of non-binding short ssDNA in DNA condensates by FRAP

The diffusion coefficients of invader (m*) within the invaded and non-invaded condensates cannot be directly determined due to the specific binding interactions between m*/m. Thus, we used a non-binding dummy strand of 20 nt labelled by Cy5 (Cy5-20nt-dummy). To measure the diffusion coefficients of Cy5-20nt-dummy in DNA condensates before invasion, we prepared a solution containing 100-times-diluted p(A_20_-m)_*n*_ DNA condensates and 10 μM of Cy5-20nt-dummy in a TE buffer with 50 mM of MgCl_2_. For the diffusion coefficients of Cy5-20nt-dummy in DNA condensates after invasion, we prepared a solution containing 100-times-diluted p(A_20_-m/m*)_*n*_ condensates, (complete m* invasion was reached before the FRAP experiments; shell labelled with k*-Atto488) and 10 μM of Cy5-20nt-dummy in a TE buffer with 50 mM of MgCl_2_.

We used the point-bleaching method (Extended Data Fig. 4). Several prebleaching images were first recorded, before bleaching by a 638-nm laser with 100% intensity at a single pixel for 3 s. Then, the post-bleaching images were recorded with the maximum frame rates over 1 min. Cross-sectional line profiles crossing the bleached centre in the post-bleaching images were taken over time, giving Gaussian distributions of the fluorescence intensity. Fitting the line profiles with a Gaussian function yielded a squared standard deviation (s.d.) of Gaussian distribution *σ*^2^, which changed over time. The diffusion coefficients (*D*) were then extracted by plotting *σ*^2^ over time with the slope being 2*D* (ref. ^63^).

### Diffusion of non-charged PNA invader into DNA condensates

The diffusion of PNA invaders (m*_PNA_-Atto488) was investigated by diluting 1 μl of the stock solution of non-fluorescent DNA condensates (diluted five times, in 50 mM of MgCl_2_) in 20 μl of solution containing 5 μM of m*_PNA_-Atto488 invader for 50 mM of MgCl_2_ in a TE buffer. The diffusion process was recorded with CLSM (Extended Data Fig. 5b). The DNA condensates were finally diluted 100 times (corresponding to 0.4 μM of m barcode), so that the ratio of Atto488-m*_PNA_/m was 12.5/1. FRAP experiments were performed on p(A_20_-m_Atto594_)_*n*_ chains of the fully invaded DNA condensates by a PNA invader with the ROI bleaching methods described above (Extended Data Fig. 5d,e).

### Cloud point temperature measurement of DNA polymers

Here 0.5 g l^–1^ of p(A_20_-m)_*n*_ was prepared in 10 μl of TE buffer and heated at 95 °C for 15 min. The cleaved p(A_20_-m)_*n*_ was either mixed with a stoichiometric amount of m*Atto488 (PNA or DNA invader) or dissolved alone at a concentration of 0.04 g l^–1^ (3.2 μM) in a TE buffer with 50 mM of MgCl_2_. Temperature-dependent ultraviolet–visible spectra were measured from 20 °C to 80 °C (1 °C min^–1^) and the onset of absorbance increase at 350 nm was used to determine the cloud point temperature.

### Enzymatic cleavage of p(A_20_-m)_*n*_ DNA polymers inside the DNA condensates to reduce the chain length for modulation of the front peak during ballistic wave diffusion

Since the temperature-induced coacervation of p(A_20_-m)_*n*_ is a molecular-weight-dependent phenomenon, it is not possible to cleave the p(A_20_-m)_*n*_ before condensate formation as this could lead to a preferred incorporation of long p(A_20_-m)_*n*_, which is unwanted. Therefore, we developed a multistep protocol to cleave the p(A_20_-m)_*n*_ chains after DNA condensate formation (Extended Data Fig. 7). The process uses enzymatic cleavage with a restriction enzyme (MluCI) that recognizes restriction sites generated by hybridization between p(A_20_-m)_*n*_ and a specifically designed cleaving strand (c*). c* contains a 10-nt sequence that can hybridize to p(A_20_-m)_*n*_, generating restriction sites for the MluCI enzyme, and another 5-nt spacer sequence attached with fluorophores (FITC) at the 5′ end as the reporter for hybridization and subsequent melting events once cleaved. Briefly, c* hybridizes to A_20_-m, the MluCI cleaves A_20_-m/c* at its restriction site and then the shorter c* fragments melt away due to their low *T*_m_ values.

For this process, the condensates first need to be made dynamic at low [Mg^2+^] (for the enzyme processing as well as for homogeneous mixing with c* within the condensates) and later made in the arrested state again at high [Mg^2+^]. To perform the enzymatic cleavage of p(A_20_-m)_*n*_ chains within DNA condensates, we first diluted 10 μl of stock solution of non-fluorescent DNA condensates (diluted five times, in 15 mM of MgCl_2_) into 20 μl of solution containing either 0.1 μM or 0.2 μM of c* (2.5 mol% or 5 mol% relative to 4 μM of A_20_-m repeat concentration in the total solution, respectively) at 15 mM of MgCl_2_ in a TE buffer. After 3 h of incubation at room temperature with gentle shaking (300 rpm) for the complete binding of c* into the DNA condensates, 2 μl of the reaction mixture was then diluted into 10 μl of solution containing 0.5 U μl^–1^ of MluCI restriction enzyme at 15 mM of MgCl_2_ in a TE buffer for subsequent restriction overnight at room temperature. The final enzyme (U) to p(A_20_-m)_*n*_ (μg) ratio was 50 U μg^–1^. MluCI recognizes a part of the hybridization domain between c* and p(A_20_-m)_*n*_ and cleaves the p(A_20_-m/c*)_*n*_ chain at this position. After cleavage, both fragments (c*′ and c*″) of the restricted c* only have five base pairs with p(A_20_-m)_*n*_ and, thus, dissociate due to their low *T*_m_ values. This was confirmed by the disappearance of the fluorescence of c*(-FITC) in the condensates (Extended Data Fig. 7b,c). The amount of cleaved sites, and hence the statistical shortening of p(A_20_-m)_*n*_, depends on the amount of c* added. Afterwards, the [Mg^2+^] of the 10 μl of the reaction mixture was further adjusted by adding 0.8 μl of TE buffer containing 500 mM of MgCl_2_ to get a final [Mg^2+^] of 50 mM. The total 10.8-μl mixture was then stored for at least 1 week for equilibration.

To test the front shape of ballistic wave diffusion, the enzymatically cleaved DNA condensates, in equilibrium with [Mg^2+^], were further diluted into a solution containing 1 μM of m*-Atto488, 0.25 μM of k*-Atto647 in a TE buffer with a total solution volume of 20 μl at 50-mM MgCl_2_ in a 384-well plate with 10–20 μl of hexadecane covering it to prevent evaporation. The DNA condensates were, thus, diluted 100 times with m barcode concentration at 0.4 μM in the total solution. The invasion process was then recorded by CLSM (Fig. 4c).

A direct quantification of the p(A_20_-m)_*n*_ chain length in cleaved DNA condensates by gel electrophoresis is not possible due to the existence of p(T_20_-k)_*n*_ in the system, which can associate with p(A_20_-m)_*n*_ and interfere with the mobility of p(A_20_-m)_*n*_ chains in gel electrophoresis. Thus, we prepared 20-μl reference solutions containing 0.05 g l^–1^ of p(A_20_-m)_*n*_ (heated previously at 95 °C totally for 30 min, same as the p(A_20_-m)_*n*_ used for preparing the DNA condensates) with 2.5 mol% and 5 mol% of c* in a TE buffer at 15 mM of MgCl_2_ incubated at room temperature for 3 h. Subsequently, 5 μl of 10 U μl^–1^ of MluCI was added to reach the same enzyme (U) to p(A_20_-m)_*n*_ (μg) ratio (50 U μg^–1^) for overnight cleavage at room temperature. These samples, thus, had the same c* to A_20_-m ratio and the same enzyme to p(A_20_-m)_*n*_ ratio as in the condensates. The samples were then characterized by gel electrophoresis (2 wt% of agarose gel, 5 V cm^–1^, 150 min in a TAE buffer containing 40 mM of Tris-HCl, 20 mM of acetic acid and 1 mM of EDTA, post-staining by SYBR Gold) to quantify the cleaved chain length compared with the chain length of the non-cleaved p(A_20_-m)_*n*_ chains (Extended Data Fig. 7d–f). The chain length distribution and average chain length of cleaved and non-cleaved p(A_20_-m)_*n*_ were calculated from the gel electrophoresis result using a previously reported method^61,62^.

### Determination of the swelling ratio of DNA condensates in different salinities

DNA condensates composed of p(A_20_-m_Atto594_)_*n*_ and p(T_20_-k_Atto488_)_*n*_ were prepared at 50 mM of MgCl_2_. Then, 1 μl of stock solution of DNA condensates (diluted five times, in 50 mM of MgCl_2_) were diluted to lower MgCl_2_ concentrations, ranging from 15 mM to 50 mM in a TE buffer with a total volume of 20 μl, yielding an m barcode concentration of 0.4 μM in the solution. The sizes of several condensates were recorded every 24 h for a total time of 120 h. The size of each condensate was then normalized to its initial size to determine the final swelling ratio in equilibrium after 120 h (Fig. 4e).

### Tuning the diffusion mechanism of invaders into DNA condensates

DNA condensates composed of p(A_20_-m_Atto594_)_*n*_ and p(T_20_-k)_*n*_ prepared at 50 mM of MgCl_2_ were diluted by five times to individual MgCl_2_ concentrations (15–50 mM). Diluted DNA condensates were then stored as stock at 4 °C for at least 1 week to reach equilibrium. For experiments with [MgCl_2_] ≥ 20 mM, 19 μl of the solution containing m*-Atto488 (1 μM) in the given MgCl_2_ concentration in a TE buffer was prepared, followed by the addition of 1 μl of DNA condensate stock solution (diluted five times). The 20-μl solution was then added into a 384-well plate with another 10–20 μl of hexadecane added on top of the solution to prevent evaporation during experiments. The final DNA condensates were, thus, diluted 100 times with an m barcode concentration of 0.4 μM in the total solution. The diffusion of m*-Atto488 into the DNA condensates was then recorded by CLSM (Fig. 4d). For experiments with MgCl_2_ below 20 mM, 1 μl of DNA condensate stock solution was first diluted to 19 μl of TE buffer containing the given MgCl_2_ concentration and transferred into a 384-well plate. Then, a small amount of concentrated m*-Atto488 was added into the same well, so that the overall m*-Atto488 concentration reached 1 μM in the mixture. Then, 10–20 μl of hexadecane was added on top to prevent evaporation. DNA condensates, which were far away from the concentrated m*-Atto488 spot, were chosen for the observation of the rapid diffusion process. This method is necessary to allow enough time for setting up imaging for the rapid diffusion process at low MgCl_2_. Direct dilution of DNA condensates into the mixture of invaders resulted in excessively fast completion of the invasion process before transferring and observing under CLSM.

### BCARS microscopy

Raman fingerprint measurements were conducted using a custom-built BCARS microscope, extensively described elsewhere^64^. Briefly, pump–probe and Stokes pulses were generated in a dual-output subnanosecond laser source (CARS-SM-30, Leukos), spatially and temporally overlapped at the sample plane of an inverted microscope (ECLIPSE Ti-U, Nikon). These pulses were then tightly focused onto a sample using a 0.85-numerical-aperture air objective (LCPlan N, Olympus). The samples were prepared by adding 1 µl of condensate solution (diluted five times, at individual MgCl_2_ concentrations) into 19 µl of TE buffer with the same concentration of MgCl_2_. The 20-µl samples were placed within a sealed channel created by attaching double-sided tape strips between a glass coverslip and a glass slide and positioned with the coverslip facing the objective. The BCARS signal was filtered from the excitation pulses and focused onto the slit of a spectrograph (Shamrock 303i, Andor), which disperses the spectral components on a cooled charge-coupled device camera (Newport DU920P-BR-DD, Andor). A piezo stage (Nano-PDQ 375 HS, Mad City Labs) was used to raster scan the samples. Data acquisition was controlled using LabView 2015 (National Instruments) software, and its processing was carried out using IgorPro (WaveMetrics). The Raman-like spectra were obtained through a modified Kramers–Kronig transform^65^, and background phase removal was performed using a Savitzky–Golay filter with a third-order polynomial along a B-spline baseline.

### DNAzyme-catalysed RNA cleavage in DNA condensates

DNA condensates containing 80% m barcode and 20% q barcode (obtained by using a mixture of p(A_20_-m)_*n*_ and p(A_20_-q)_*n*_ during condensate formation) were used for DNAzyme^56^ encapsulation by q/q* hybridization. Here 5 μl of DNA condensates (1.6 μM of q barcode and 6.4 μM of m barcode) were mixed with 1 μl of q*-DNAzyme (8 μM) and 2 μl of m* (50 μM) into a total of 12 μl of TE buffer containing 50 mM of MgCl_2_ for 1 h for complete DNAzyme encapsulation and invasion-induced arrested-to-dynamic transition of DNA condensates. For arrested DNA condensates loaded with DNAzyme, the same solution was prepared, except m* was not added. A substrate solution was prepared by mixing 2 μl of p-A_1000_ (2 μM, synthesized by terminal deoxynucleotidyl transferase^31^), 2 μl of p*-substrate (400 nM) and 1 μl of RNase inhibitor (40 U μl^–1^ (Thermo Scientific)) into a total of 8 μl of TE buffer containing 50 mM of MgCl_2_ for 1 h for complete hybridization between p-A_1000_ and p*-substrate. Then, the substrate solution was introduced into the solution containing arrested or dynamic DNAzyme-loaded DNA condensates. The reaction was monitored under CLSM (Extended Data Fig. 10).

### AFM measurements

To perform force spectroscopy on condensates, we added condensates (before or after invasion, in a TE buffer containing 50 mM of MgCl_2_) into an AFM liquid cell on a glass slide on top of the stage of the inverted microscope. The AFM colloid probe (borosilicate glass bead diameter = 18.2 ± 1 μm; spring constant = 0.6 N m^–1^) was then approached to the top of a condensate under the microscope. The force spectroscopy measurement was then conducted in the contact mode. For each condensate type, we measured over five force spectroscopy curves in a compression–retraction cycle, and the hysteresis is quantified by integrating the area within the compression–retraction curves.

### Statistical analysis

All data points featuring statistical analysis are presented as mean ± s.d. or mean ± standard error. The sample size (*n*) for each statistical analysis is reported in the relevant figure legend. FRAP, FLIM and AFM measurements were performed on several condensates from two independent samples. Diffusion mechanisms tuned by chain length and salinity were measured on several condensates from two independent samples. BCARS measurements were performed on several condensates from three independent samples. Titration and swelling experiments were carried out on several condensates from a series of samples at each condition. Diffusion kinetics were measured from four independent samples at each condition. *P* values are calculated by one-way analysis of variance test. Statistical analysis was carried out using Origin 2023 (v10.0.0.154) and Microsoft Excel (Version 2307).

### Reporting summary

Further information on research design is available in the Nature Portfolio Reporting Summary linked to this article.

## Online content

Any methods, additional references, Nature Portfolio reporting summaries, source data, extended data, supplementary information, acknowledgements, peer review information; details of author contributions and competing interests; and statements of data and code availability are available at 10.1038/s41565-025-01941-0.

## Supplementary information

Supplementary Information Supplementary Methods 1–6, Discussion 1, Figs. 1–4 and Tables 1 and 2.

Reporting Summary

Supplementary Video 1 Ballistic wave diffusion process of invader strands into a DNA condensate. In contrast to Fickian diffusion, the ballistic wave diffusion observed here shows an ultrasharp diffusion front with the highest intensity. The front propagates linearly with time accompanied with the swelling of the DNA condensate.

Supplementary Video 2 Ballistic wave diffusion process of invader strands into a DNA condensate with an in-chain label.

Supplementary Video 3 Diffusion mechanism at different [Mg^2+^] values. The diffusion adapts from ballistic wave diffusion to ballistic front diffusion and finally to Fickian diffusion with a decrease in [Mg^2+^].

Supplementary Video 4 Ballistic wave diffusion process of invader strands into a membraneless DNA condensate featuring the sticker–spacer model.

Supplementary Data 1 Statistical source data.

Supplementary Data 2 Sequences of oligonucleotides and DNA polymers used.

## Source data

Source Data Fig. 1 Statistical source data.

Source Data Fig. 2 Statistical source data.

Source Data Fig. 3 Statistical source data.

Source Data Fig. 4 Statistical source data.

Source Data Extended Data Fig. 1 Statistical source data.

Source Data Extended Data Fig. 2 Statistical source data.

Source Data Extended Data Fig. 4 Statistical source data.

Source Data Extended Data Fig. 5 Statistical source data.

Source Data Extended Data Fig. 6 Statistical source data.

Source Data Extended Data Fig. 7 Statistical source data.

Source Data Extended Data Fig. 8 Statistical source data.

Source Data Extended Data Fig. 9 Statistical source data.

Source Data Extended Data Figs. 1 and 7 Unprocessed gels.

## Data Availability

All data supporting the findings of this study are presented in the Article and its Supplementary Information. Source data are provided with this paper.

## Code availability

The code for reaction-diffusion simulation and oxDNA MD simulation is available via GitHub at https://github.com/WaltherLab/reaction-diffusion-in-biomolecular-condensates.
