# Supplementary material for: Ballistic diffusion fronts in biomolecular condensates
Source: Nat Nanotechnol. 2025 Jun 6;20(8):1062–70. doi: 10.1038/s41565-025-01941-0 (PMC12373509; doi:10.1038/s41565-025-01941-0)
Supplement: Supplementary file 1 — Supplementary Methods 1–6, Discussion 1, Figs. 1–4 and Tables 1 and 2. [file 41565_2025_1941_MOESM1_ESM.pdf]

nature nanotechnology

Supplementary information https://doi.org/10.1038/s41565-025-01941-0

Ballistic diffusion fronts in biomolecular condensates

In the format provided by the authors and unedited

Supplementary Information

Ballistic diffusion fronts in biomolecular condensates

Weixiang Chen[1], Brigitta Dúzs[1], Pablo G. Argudo[2], Sebastian V. Bauer[1], Wei Liu[1], Avik Samanta[1], Sapun H. Parekh[2,3], Mischa Bonn[2], Andreas Walther[1]*

[1]Life-Like Materials and Systems, Department of Chemistry, University of Mainz; Mainz, 55128, Germany.

[2]Department of Molecular Spectroscopy, Max Planck Institute for Polymer Research; Mainz, 55128, Germany.

[3]Department of Biomedical Engineering, University of Texas at Austin; Austin, TX, 78712, United States.

*Corresponding author. Email: andreas.walther@uni-mainz.de

Table of Contents

Supplementary Method 1. Molecular Dynamics (MD) simulations using oxDNA 3

Supplementary Method 2. Numerical model for the simulation.. 5

Supplementary Method 3. Melting temperature estimation 7

Supplementary Method 4. Finite element simulation with COMSOL 7

Supplementary Method 5. Determination of m barcode concentration inside DNA condensates . 7

Supplementary Method 6. Determination of Mg^{2+} concentration inside and outside DNA condensates 7

Supplementary Discussion 1. Parameter sweeps in simulations to derive the influence of differences in diffusion coefficients on the appearance of ballistic wave diffusion....................... 9

Supplementary Figure 1. Individual force spectroscopy on condensates before and after invasion. 10

Supplementary Figure 2: Determination of the m barcode concentration in DNA condensate by titration.. 11

Supplementary Figure 3. Acceleration of front propagation at the end stage for 2D and 3D simulations... 12

Supplementary Figure 4. Comparison of Mg^{2+} concentrations inside and outside condensates.. 13

Supplementary Table 1. General parameters used in the reaction-diffusion simulations............. 14

Supplementary Table 2. Specific parameter sets for reaction-diffusion simulation results in different figures.. 15

Supplementary Method 1. Molecular Dynamics (MD) simulations using oxDNA

Theoretical predictions for the change in rigidity of $p(A_{20}\text{-}m)_n$ upon hybridization with invader strands m* and corresponding expansion were calculated on the basis of the oxDNA model[34,66,67]. End-to-end distances were calculated for (i) a ssDNA $p(A_{20}\text{-}m)_n$ with $n = 25$ repetition units and (ii) its partly hybridized dsDNA $p(A_{20}\text{-}m/m^*)_n$ counterpart hybridized with 25 invader strands m*. Molecular dynamics (MD) simulations were performed with the standalone version of oxDNA (v3.6.0) using oxDNA2 interactions with sequence-dependent parameters as provided in the standalone version. Accounting for sequence dependency is advisable to study the mechanics of our system because poly(A) has been found to be exceptionally straight and stiff[68]. Results were analyzed with oxDNA analysis tools included in the standalone version, and with a custom-made ready-to-use tool that determines radii of gyration (R_g) from oxDNA data.

1. Simulation Setup

All simulations were performed within the canonical ensemble with temperature $T = 20$ °C. A Bussi-Donadio-Parrinello thermostat was used during the relaxation period and a Johnson thermostat during data collection, with a diffusion coefficient of 2.5 simulation unit (s.u). and 103 Newtonian steps (steps during which the system evolves according to Newton's equations of motions, before a random subset of particle momenta is sampled from a Maxwell distribution for thermostating). The skin parameter for Verlet lists was set to 0.1. Interactions were always calculated for oxDNA interaction type DNA2 using sequence-dependent parameters provided by the oxDNA standalone version.

Since Mg^{2+} is not included in the oxDNA model, we adjusted ionic strength by setting $[Na^+] = 1$ M as recommended by Doye et al.[69] for establishing representative conditions for DNA systems operating at $[Mg^{2+}] = 12.5$ mM. Using this value is further justified by the fact that, while the related quantity of persistence length depends on salt concentrations, $[Na^+] = 1$ M fits very well $[Mg^{2+}] = 50$ mM in the case of a 1201 bp dsDNA[48].

1.1 Initialization

Initial configurations were generated using the tool `generate-sa.py` included in the standalone version of oxDNA with box lengths set to $L = 500$ s.u.. The initial configuration of the hybridized dsDNA was derived from the one of a hypothetical full dsDNA by manual deletion of T_{20} parts that would be complementary to the unhybridized A_{20} parts of the dsDNA.

1.2 Relaxation

To relax the initial configurations, we followed the Rovigatti protocol[70]: As first stage, a short MC run (10^3 steps) with limited backbone forces was performed to account for the possibility of an unphysically stressed initial configuration. As second stage, an MD simulation with limited backbone forces (3.1×10^7 steps, $\Delta t = 10^{-3}$ s.u.) followed up. In the case of ssDNA, the simulation box lengths had to be reduced to $L = 200$ s.u. after 10^6 steps, because the maximum number of particles per cell grew too large due to the pronounced contraction of ssDNA compared to the initial configuration. We added another MD simulation with unlimited backbone interactions (4×10^7 steps, $\Delta t = 10^{-3}$ s.u.) as third stage for relaxation until changes in both total and hydrogen-bond energies had reduced to thermal fluctuations.

1.3 Data collection

MD simulations were performed on a GPU for 2×10^9 steps with a step size of $\Delta t = 5 \times 10^{-3}$ s.u.. Configurations and energies were evaluated every 10^5 steps, giving a sample size of $N = 2 \times 10^4$ configurations for each model DNA. On an NVIDIA GeForce RTX 3060 Ti, this took about 2.8 days in the case of ssDNA and 3.3 days in the case of our hybridized dsDNA (wall time).

2. Data analysis

2.1 Analysis tool for gyration analysis

A command line tool was implemented in Python 3 to calculate radii of gyration, R_g, from oxDNA topology and trajectory files. It is available via GitHub repository (https://github.com/WaltherLab/reaction-diffusion-in-biomolecular-condensates) as `gyration.py` and can readily be used on any data from oxDNA simulations (provided in the "traditional" oxDNA format).

2.2 Calculations

The mean squared radius of gyration $\langle R_g^2 \rangle$ were calculated both in a sequence-averaged and a sequence-dependent way, thereby treating the analyzed nucleotides as monomeric units in a homopolymer or a heteropolymer, respectively. For a homopolymer with N monomeric units at positions $\boldsymbol{r}_i$, the root mean square radius of gyration was calculated as

$$\langle R_g{}^2 \rangle^{1/2} = \sqrt{\left\langle \frac{1}{N} \sum_{i=1}^{N} (r_i - r)^2 \right\rangle},$$

where $\langle \quad \rangle$ is the ensemble average and $r = \frac{1}{N} \sum_{i=1}^{N} r_i$ is the mean particle position. In this case, $\langle R_g^2 \rangle$ and mean square end-to-end distance ($\langle h^2 \rangle$) are related via $\langle R_g^2 \rangle = \langle h^2 \rangle / 6$ relationship[71]. Thus, the root mean square end-to-end distance ($\langle h^2 \rangle^{1/2}$) is calculated, too. For a heteropolymer, the root mean square radius of gyration was calculated as

$$\langle R_g{}^2 \rangle^{1/2} = \sqrt{\left\langle \frac{1}{M} \sum_{i=1}^{N} m_i \, s_i^2 \right\rangle},$$

where, for a certain nucleotide i, m_i is its mass, $M = \sum_{j=1}^{N} m_j$ the total mass of all nucleotides, and $s_i = \left| r_i - \frac{1}{M} \sum_{j=1}^{N} m_j \, r_j \right|$ is its distance from the common centre of mass[72].

Supplementary Method 2. Numerical model for the simulation

The reaction-diffusion model implements two steps, i.e., binding (R1) and swelling (R2), and is based on the following stoichiometric and rate equations:

$$\mathrm{m}^* + \mathrm{m} \rightarrow \mathrm{M}_{\mathrm{arrested}} \quad \text{(R1)}$$

$$r_1 = k_{\mathrm{bind}}[\mathrm{m}^*][\mathrm{m}] \quad (1)$$

$$f_{\mathrm{swell}}\,\mathrm{M}_{\mathrm{arrested}} \rightarrow \mathrm{M}_{\mathrm{swollen}} \quad \text{(R2)}$$

$$r_2 = k_{\mathrm{swell}}[\mathrm{M}_{\mathrm{arrested}}], \quad (2)$$

Where m* is the invader strand diffusing from outside, m is the ssDNA barcode inside the condensate, $\mathrm{M}_{\mathrm{arrested}}$ and $\mathrm{M}_{\mathrm{swollen}}$ are the hybridized dsDNA forms of m/m* in the arrested state and in the dynamic state, respectively. The f_{swell} stoichiometric coefficient in (R2) mimics the volume expansion. Molar concentrations are indicated by [ ], k_{bind} and k_{swell} are reaction rate coefficients.

The spatiotemporal behavior of the condensate is described by following set of equations in 1D space with the spatial coordinates $x \in \{0, \ldots, L\}$, where $x = 0$ corresponds to the condensate/outer medium interface and $x = L$ is the centre of the condensate (Extended Data Fig. 6a):

$$\partial_t[\mathrm{m}^*] = -\,k_{\mathrm{bind}}[\mathrm{m}^*][\mathrm{m}] + D_{\mathrm{m}^*}\Delta[\mathrm{m}^*] \quad (3)$$

$$\partial_t[\mathrm{m}] = -\,k_{\mathrm{bind}}[\mathrm{m}^*][\mathrm{m}] + D_{\mathrm{m}}\Delta[\mathrm{m}] \quad (4)$$

$$\partial_t[\mathrm{M}_{\mathrm{arrested}}] = k_{\mathrm{bind}}[\mathrm{m}^*][\mathrm{m}] - f_{\mathrm{swell}}\,k_{\mathrm{swell}}[\mathrm{M}_{\mathrm{arrested}}] + D_{\mathrm{M}_{\mathrm{arrested}}}\Delta[\mathrm{M}_{\mathrm{arrested}}] \quad (5)$$

$$\partial_t[\mathrm{M}_{\mathrm{swollen}}] = k_{\mathrm{swell}}[\mathrm{M}_{\mathrm{arrested}}] + D_{\mathrm{M}_{\mathrm{swollen}}}\Delta[\mathrm{M}_{\mathrm{swollen}}] \quad (6)$$

In our numerical model, for simplicity, m* is considered to be the only diffusively transported component with the diffusion coefficient D_{m^*}, whereas $D_{\mathrm{m}} = D_{\mathrm{M}_{\mathrm{arrested}}} = D_{\mathrm{M}_{\mathrm{swollen}}} = 0$.

At the definition of D_{m^*} we apply a deviation from the classical Fick's second law: D_{m^*} depends on the local state (arrested or swollen) of the condensate. The state is defined by comparing the local $[\mathrm{M}_{\mathrm{swollen}}]$ with a threshold value $[\mathrm{M}_{\mathrm{swollen}}]^{\mathrm{thres}}$.

$$D_{\mathrm{m}^*} = \begin{cases} D_0, & \text{if} \quad [\mathrm{M}_{\mathrm{swollen}}] < [\mathrm{M}_{\mathrm{swollen}}]^{\mathrm{thres}} \quad \text{arrested state} \\ D_1, & \text{if} \quad [\mathrm{M}_{\mathrm{swollen}}] \geq [\mathrm{M}_{\mathrm{swollen}}]^{\mathrm{thres}} \quad \text{dynamic state.} \end{cases} \quad (7)$$

In simple terms, this governs whether m* diffuses quickly in already dynamic/invaded parts, or whether it diffuses slowly in arrested/non-invaded parts. Dirichlet boundary conditions are applied at the condensate surface, i.e., $[\mathrm{m}^*]_{x=0} = [\mathrm{m}^*]_{\mathrm{i}}$, and no-flux boundary conditions at the inner side of the condensate, i.e., $\partial_x[\mathrm{m}^*]_{x=L} = 0$. The initial conditions are $[\mathrm{m}^*]_{t=0} = 0$, $[\mathrm{m}]_{t=0} = [\mathrm{m}]_{\mathrm{i}}$, and $[\mathrm{M}_{\mathrm{arrested}}]_{t=0} = [\mathrm{M}_{\mathrm{swollen}}]_{t=0} = 0$ at $x = 1, \ldots, L$. Here [ ] is the space- and time-dependent concentration in the condensate, ∂_t is the time derivative, and $\Delta = \frac{\partial^2}{\partial x^2}$ is the Laplace operator. The parameter values are listed in Supplementary Table 1 and 2.

The partial differential equations were discretized with standard second order finite difference scheme, and the resulting systems were solved by the SUNDIALS CVODE solver using backward differentiation formula method[73]. In Fig. 3b, the mesh size was 400, the grid spacing was $\Delta x = 0.05\ \mu\mathrm{m}$, the time step was $\Delta t = 0.001\ \mathrm{s}$, the absolute and relative error tolerances were 10^{-10} and 10^{-12}, respectively. In Fig. 4a,b the mesh size was 7000, the grid spacing was $\Delta x =$

0.005 µm, the time step was $\Delta t = 0.005$ s, the absolute and relative error tolerances were 10^{-6} and 10^{-12}, respectively. In Fig. 4j,l the mesh size was 75000, the grid spacing was $\Delta x = 0.006$ µm, the time step was $\Delta t = 0.01$ s , the absolute and relative error tolerances were 10^{-5} and 10^{-8}, respectively. In Extend Data Fig. 7c-e, the mesh size was 7000, the grid spacing was $\Delta x = 0.005$ µm, the time step was $\Delta t = 0.005$ s, the absolute and relative error tolerances were 10^{-6} and 10^{-12}, respectively.

The front position $x_\mathrm{f}(t)$ is determined as the x, where the $I_t(x) = [\mathrm{m}^*]_t(x) + [\mathrm{M}_\mathrm{arrested}]_t(x) + [\mathrm{M}_\mathrm{swollen}]_t(x)$ curve has its maximum. It is in accordance with the experiments, where these species correspond to the detectable fluorescence. The front velocity (v) is determined by linear fitting of the $x_\mathrm{f}(t)$ data:

$$x_\mathrm{f}(t) = v_0 + vt, \quad (8)$$

where $v_0 = 0$. The width of the front (W) is the full width at half maximum (FWHM) of the $I_t(x)$ curve. The half maximum value is defined as $I_{t,\mathrm{HM}} = \frac{I_t(x_\mathrm{f}) + I_t(x_0)}{2}$, where $I_t(x_0)$ indicates the fluorescence at the equilibrium swollen region backwards the front (Fig. 4h).

The simulation code is available at https://github.com/WaltherLab/reaction-diffusion-in-biomolecular-condensates.

Supplementary Method 3. Melting temperature estimation

All melting temperatures (T_ms) were estimated by OligoAnalyzer Tool (IDT) with experimental conditions as input for oligonucleotide concentration and [Mg^{2+}].

The m barcode concentration inside DNA condensates was determined in Supplementary Figure 2 and used for the determination of important T_ms as follows: For T_m of m/m* (71.5 °C), we applied 200 µM (note that there is swelling induced by m/m* hybridization) as the oligonucleotide concentration, and 50 mM Mg^{2+} for [Mg^{2+}].

For melting points of c*/p(A_{20}-m)$_n$ (40.2 – 44.4 °C, before cleavage), c*'/p(A_{20}-m)$_n$ (0 °C, after cleavage), and c*''/p(A_{20}-m)$_n$ (3.4 – 11.6 °C, after cleavage), we applied 10, 20, and 40 µM as the oligonucleotide concentration. Since the DNA condensates swell by ca. 2.2-fold in volume at 15 mM $MgCl_2$ (Fig. 4e), the m barcode concentration in the condensates is roughly 400 µM. The applied concentrations correspond to experimentally used 2.5, 5, 10 mol% c* relative to m-barcode concentration in the condensates. We apply 15 mM Mg^{2+} for [Mg^{2+}].

Supplementary Method 4. Finite element simulation with COMSOL

We used COMSOL Multiphysics 5.6 to model ballistic wave diffusion in different dimensions. We combined two modules in COMSOL, i.e., **Chemistry** and **Transport of Diluted Species** for simulations. We used the same input as our numerical model and the inputs are summarized in Supplementary Table 2. We used here Heaviside function for the change of diffusion coefficients at different regions for consistency. The geometry is a rectangle (1 × 20 μm^2) for 1D simulation, a circle (radius = 20 µm) for 2D simulation, and a sphere (radius = 20 µm) for 3D simulation. For 1D simulation, we applied Dirichlet boundary conditions with [m*] = 1 at the left side of the rectangle, while for 2D and 3D simulation we applied Dirichlet boundary conditions with [m*] = 1 at the circumference of the circle and surface of the sphere to induce invasion process.

Supplementary Method 5. Determination of m barcode concentration inside DNA condensates

We used a titration method to measure the concentration of the m barcode in the p(A_{20}-m)$_n$ DNA condensates (Supplementary Figure 2). Non-fluorescent DNA condensates at 50 mM $MgCl_2$ were 100 times diluted (0.4 µM barcode in total solution) in a 20 µL solution containing different concentrations of m*-Atto488 ranging from 50 to 400 µM at 50 mM $MgCl_2$ in TE buffer. We confirmed complete invasion and swelling of the p(A_{20}-m)$_n$ DNA condensates before taking CLSM images for the quantification of fluorescence intensity inside (I_{in}) and outside (I_{out}) of the condensates. The corresponding intensity ratio ($R = I_{in}/I_{out}$) was plotted against the m*-Atto488 concentration and fitted to extract the m barcode concentration inside dynamic/invaded DNA condensate. By knowing the swelling during invasion, it is possible to calculate the original m barcode concentration in the pristine condensates.

Supplementary Method 6. Determination of Mg^{2+} concentration inside and outside DNA condensates

We used the Mg^{2+} indicator Mag-fluo-4 AM to determine the Mg^{2+} concentrations inside and outside the condensates before and after invasion (Supplementary Figure 4). First, we calibrated

the Mg^{2+} indicator by preparing a series of 10 μM Mag-fluo-4 AM solutions in PBS buffer with varying Mg^{2+} concentrations, confirming that fluorescence intensity increases with the Mg^{2+} concentration. Next, we verified that the presence of $p(A_{20}\text{-}m)_n$, $p(T_{20}\text{-}k)_n$, and m* does not significantly affect the fluorescence intensity of 10 μM Mag-fluo-4 AM at 2 mM Mg^{2+}, which is the relevant Mg^{2+} concentration range for our experiments (measured in dilution from an original condensate). For the condensate experiments, we prepared triplicate samples of condensates at 50 mM Mg^{2+} before and after invasion. Each sample was centrifuged, and 1 μL of both the supernatant and sediment (=condensate) was collected and diluted into 20 μL of PBS buffer containing 10 μM Mag-fluo-4 AM. The fluorescence intensities of these samples were measured with a plate reader. Excitation = 485 nm, emission = 525 nm, band width = 15 nm.

Supplementary Discussion 1. Parameter sweeps in simulations to derive the influence of differences in diffusion coefficients on the appearance of ballistic wave diffusion

Extended Data Fig. 6b shows extended parameter sweep for different diffusion coefficients in the arrested/dense interior (D_0) versus the dynamic/swollen exterior (D_1). The data helps to demonstrate that the occurrence of the ballistic diffusion front, *i.e.*, (1) sharp diffusion front and (2) linear front propagation kinetics, follows a gradual transition from Fickian diffusion based on the differences between D_0 and D_1 (See from (v) to (i)). Once the difference exceeds two orders of magnitude, the front propagation follows linear kinetics in certain length (tens of micrometers) and time scales (hundreds of minutes). In the transition period, where D_0 and D_1 only have a small difference, at long time and length scales, there will be a transition from ballistic diffusion to Fickian diffusion. This aligns with case II diffusion for solvent penetration into plastics[38]. Compare the two top lines in b (i) – (vi) – (viii) (and – (x) for very long-term simulations) versus (ii) – (vii) – (ix). Ultimately, the difference between D_0 and D_1 will determine the length and time scales of the ballistic diffusion front. In the context of biomolecular condensates, whose size are 1 ~ 10 µm, short length and time scales dominate, which therefore locates them in the regime with ballistic diffusion front.

Besides, since our model uses fast binding kinetics of the invader m* to the core $p(A_{20}\text{-}m)_n$ and a slow relaxation kinetics, there can even be a front accumulation in case of Fickian diffusion with a mild D_0 and D_1 difference (see beginning of (iii), (iv), and (v)). Critically, for all Fickian diffusion cases, the accumulation at the front vanishes over time, while the ballistic wave diffusion can maintain the front accumulation with a stable high intensity front controlled by balanced binding and relaxation kinetics.

To summarize, it is the difference between D_0 and D_1 that defines the ballistic wave diffusion in time and length scales, while the interplay between binding and relaxation kinetics determines the front accumulation.

Supplementary Figure 1. Individual force spectroscopy on condensates before and after invasion.

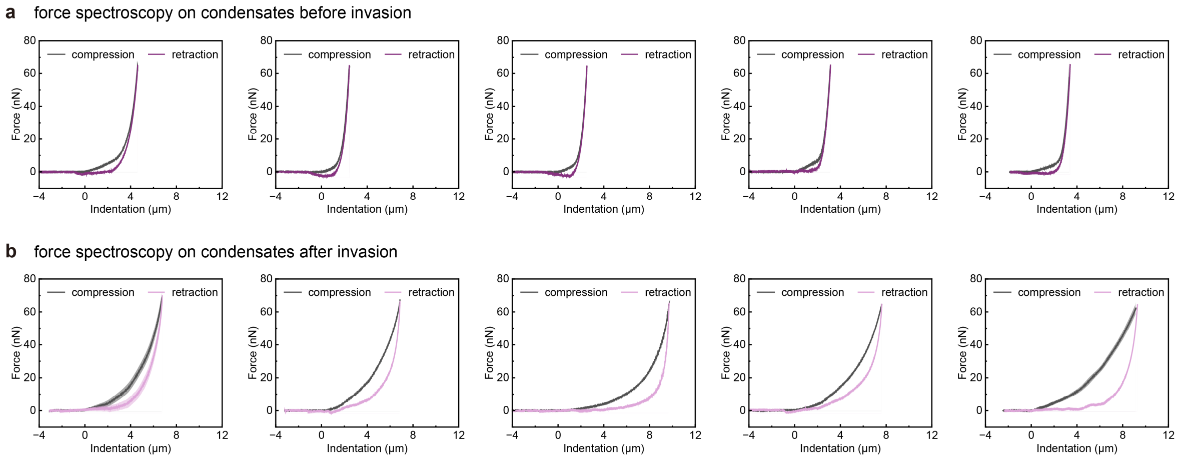

a, Individual force spectroscopy measurements on condensates *before invasion*, showing rather elastic behavior with high stiffness, and low hysteresis (mean ± s.d., N = 5 condensates measured). **b**, Individual force spectroscopy measurements on condensates *after invasion*, showing rather liquid-like behavior with low stiffness and high hysteresis (mean ± s.d., N = 5 condensates measured).

Supplementary Figure 2: Determination of the m barcode concentration in DNA condensate by titration.

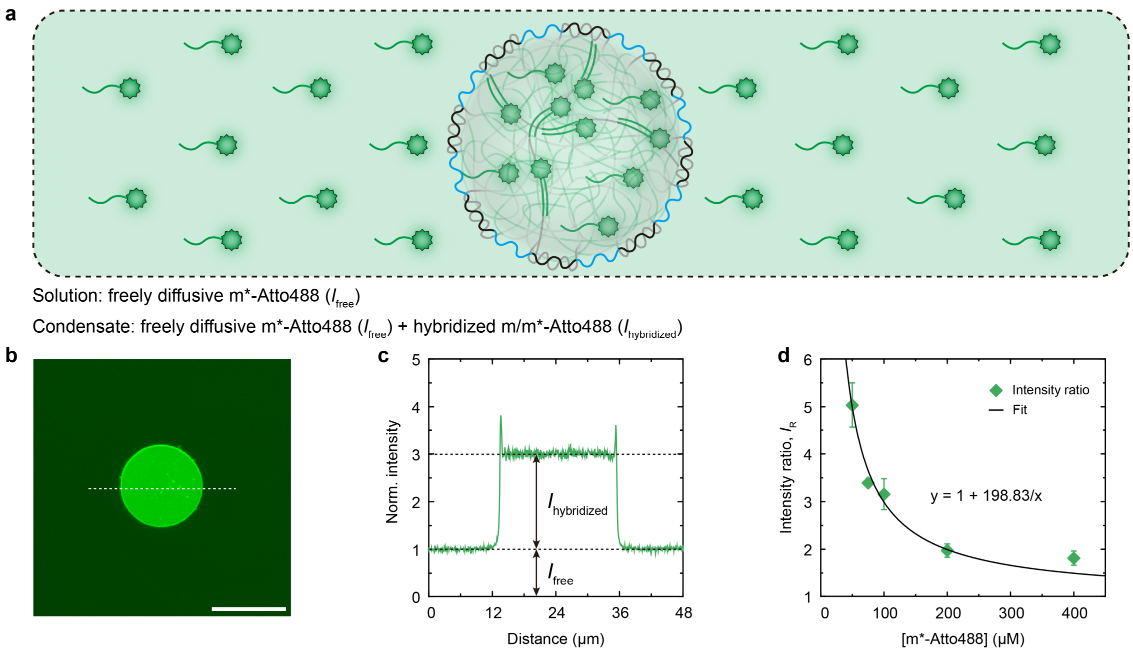

a, Schematic representation of the titration experiment for determining the m barcode concentration in DNA condensates (Supplementary Method 5). **b**, Representative CLSM image of a dynamic condensate in solution containing 100 µM m*-Atto488. **c**, Corresponding cross-sectional line profile along the dashed line in **b**. The intensity is normalized to the background solution intensity (I_{free}). Thus, normalized intensity = 1 means the intensity contributed from freely diffusive fluorophores, while the additional contributions above 1 within a DNA condensate stem from the hybridized m*-Atto488, $I_{hybridized}$. **d**, Intensity ratio (I_R) as a function of m*-Atto488 concentration (mean ± s.d., N = 6 condensates measured for 50 and 400 µM [m*-Atto488], and 4 condensates measured for other [m*-Atto488]). The scale bar is 20 µm.

Analysis and experiment method: DNA condensates were mixed with high concentrations m*-Atto488 (50 – 400 µM) with a total concentration of m barcodes in the solution at 0.4 µM (inside the condensates). During hybridization of m*-Atto488 into the condensates, the consumption of m*-Atto488 in the background solution can be neglected due to the high excess. Within the DNA condensate, all m barcodes are hybridized by m*-Atto488, and the condensate is in a dynamic and swollen state. The fluorescence intensity in the solution is only due to freely diffusing m*-Atto488, yielding I_{free}. In the condensate, there are both hybridized m*-Atto488 and freely diffusing m*-Atto488, giving $I_{hybridized} + I_{free}$. We define the intensity ratio (I_R) as the ratio between intensity inside the condensate and intensity in the background solution, which can be written as $I_R = (I_{hybridized} + I_{free})/I_{hybridized}$. We consider a linear correlation of intensity with dye concentration ($c \propto I$). Under this consideration, we can get $(c_{hybridized} + c_{free})/c_{free} = (I_{hybridized} + I_{free})/I_{free} = I_R$, which can be re-written to $c_{hybridized}/c_{free} + 1 = I_R$. c_{free} is the concentration of m*-Atto488 added in solution (known, independent variable), and I_R can be experimentally measured (dependent variable). Therefore, when performing a series of concentration-dependent experiments and measuring the corresponding I_R, it is possible to fit all data points with the equation $c_{hybridized}/c_{free} + 1 = I_R$. This yields the parameter $c_{hybridized}$, which is the concentration of hybridized m*-Atto488, and thus also the concentration of m barcode inside the condensates in the swollen state. The data points in (d) are fitted, and the hybridized concentration of m*-Atto488 is determined to be ca. 200 µM, reflecting the concentration of m barcode within the condensates after invasion and swelling. Based on a 4-fold swelling ratio in volume due to invasion (Fig. 1e), the barcode concentration can be calculated to ca. 800 µM ≈ 1 mM in the non-invaded pristine condensates.

Supplementary Figure 3. Acceleration of front propagation at the end stage for 2D and 3D simulations.

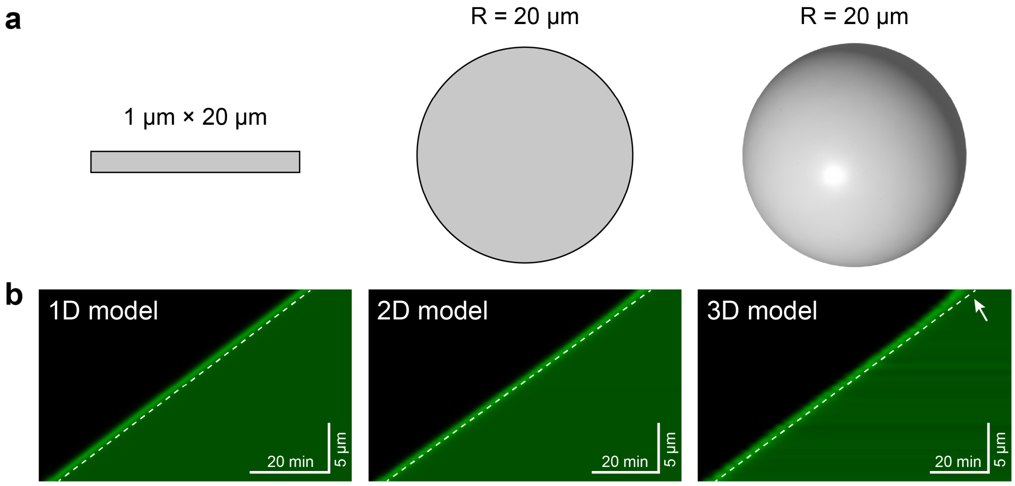

a, Geometries used for simulation in different dimensions. **b**, Simulated kymographs show the front propagation behavior of ballistic wave diffusion in different dimensions. No acceleration of front propagation in 1D, negligible acceleration in 2D, and more pronounced acceleration in 3D. The acceleration at the end stage in 3D is indicated with a white arrow.

Supplementary Figure 4. Comparison of Mg^{2+} concentrations inside and outside condensates.

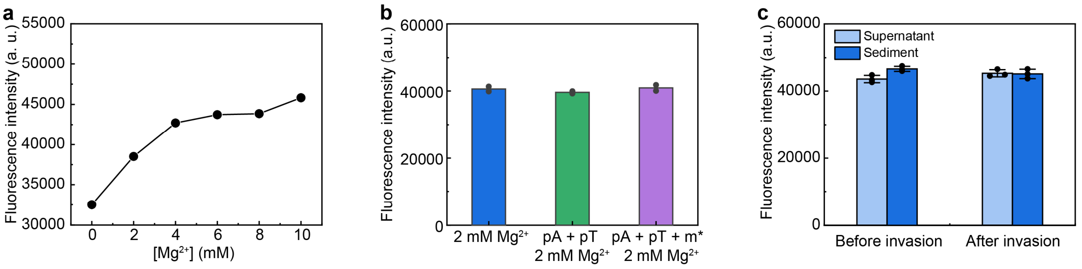

a, Calibration curve showing the fluorescence intensity of 10 μM Mg^{2+} indicator (Mag-fluo-4 AM) in PBS buffer at varying Mg^{2+} concentrations (mean ± s.d., $N = 2$ independent experiments for each [Mg^{2+}]). **b**, Fluorescence intensity of 10 μM Mg^{2+} indicator in PBS buffer with and without the presence of DNA polymers (pA and pT) and invader strand (m*) show no significant differences. Note that the concentrations of pA, pT, and m* were matched to those in the sediment samples before and after invasion in **c** (mean, $N = 2$ independent experiments for each condition). **c**, Fluorescence intensity of 10 μM Mg^{2+} indicator in 20-fold diluted supernatant and sediment samples from condensates at 50 mM Mg^{2+} before and after invasion. (mean ± s.d., $N = 3$ independent experiments for each condition). See Supplementary Method 6 for experiment details.

Parameter	Value	Unit	From
k_{bind}	100	$m^3 \cdot mol^{-1} \cdot s^{-1}$	Manual optimization
k_{swell}	Varied from 0.001 – 0.1	s^{-1}	Manual optimization
D_0	$5 \cdot 10^{-12}$	$m^2 \cdot s^{-1}$	Estimated from experiments (Extended Data Fig. 4)
D_1	$5 \cdot 10^{-9}$	$m^2 \cdot s^{-1}$	Estimated from experiments (Extended Data Fig. 4) + manual optimization
$D_{\mathrm{cons.}}$	$5 \cdot 10^{-11}$	$m^2 \cdot s^{-1}$	Between D_0 and D_1 +manual optimization
$[\mathrm{m}^*]_{\mathrm{i}}$	Varied from 0.0002 – 0.1	mM	Input
$[\mathrm{m}]_{\mathrm{i}}$	1	mM	Estimated from experiments (Supplementary Figure 2)
$[\mathrm{M}_{\mathrm{swollenl}}]^{\mathrm{thres}}$	0.24	mM	Manual optimization
f_{swell}	4	None	Estimated from experiments (Fig. 1e)

Supplementary Table 1. General parameters used in the reaction-diffusion simulations.

Figures	k_{bind} ($m^3 \cdot mol^{-1} \cdot s^{-1}$)	k_{swell} (s^{-1})	D_0 ($m^2 \cdot s^{-1}$)	D_1 ($m^2 \cdot s^{-1}$)	$[m^*]_i$ (mM)	$[m]_i$ (mM)	$[M_{swollenl}]^{thres}$ (mM)	f_{swell} (/)
**Fig. 3b**	100	0.004	$5 \cdot 10^{-12}$	$5 \cdot 10^{-9}$	0.001	1	0.24	4
**Fig. 3c**	100	0.1	$5 \cdot 10^{-12}$	$5 \cdot 10^{-9}$	0.001	1	0.24	4
**Fig. 3d**	100	0.1	$5 \cdot 10^{-11}$	$5 \cdot 10^{-11}$	0.001	1	0.24	4
**Fig. 4a, b**	100	0.001 - 0.1	$5 \cdot 10^{-12}$	$5 \cdot 10^{-9}$	0.001	1	0.24	4
**Fig. 4j, l**	100	0.004	$5 \cdot 10^{-12}$	$5 \cdot 10^{-9}$	0.0002 - 0.01	1	0.24	4
**Extended Data Fig. 6b**	100	0.004	10^{-13} - 10^{-8}	10^{-13} - 10^{-8}	0.001	1	0.24	4
**Extended Data Fig. 6c-e**	100	0.004	$5 \cdot 10^{-12}$	$5 \cdot 10^{-9}$	0.001 - 0.1	1	0.24	4
**Suppleme ntary Figure 3**	100	0.001	$5 \cdot 10^{-12}$	$5 \cdot 10^{-9}$	0.001	1	0.24	4

Supplementary Table 2. Specific parameter sets for reaction-diffusion simulation results in different figures.

References

66. Snodin, B. E. K. *et al*. Introducing improved structural properties and salt dependence into a coarse-grained model of DNA. *J. Chem. Phys.* **142**, 234901 (2015).

67. Naskar, S. & Maiti, P. K. Mechanical properties of DNA and DNA nanostructures: comparison of atomistic, Martini and oxDNA models. *J. Mater. Chem. B* **9**, 5102-5113 (2021).

68. Mitchell, J. S., Glowacki, J., Grandchamp, A. E., Manning, R. S. & Maddocks, J. H. Sequence-dependent persistence lengths of DNA. *J. Chem. Theory Comput.* **13**, 1539-1555 (2017).

69. Doye J. P. K. *et al*. The oxDNA coarse-grained model as a tool to simulate DNA origami. In: Valero, J. (eds) DNA and RNA origami. *Methods in Molecular Biology* **2639**, 93-96 (2023).

70. Rovigatti, L. Relaxing initial configurations. https://lorenzo-rovigatti.github.io/oxDNA/relaxation.html (2022)

71. Cantor, C. R. & Schimmel, P. R. *Biophysical Chemistry Part III: The behavior of biological macromolecules* p. 983 (1980).

72. Stepto, R. *et al*. Definitions of terms relating to individual macromolecules, macromolecular assemblies, polymer solutions, and amorphous bulk polymers (IUPAC Recommendations 2014). *Pure Appl. Chem.* **87**, 71-120 (2015).

73. Hindmarsh, A. C. *et al*. SUNDIALS: Suite of nonlinear and differential/algebraic equation solvers. *ACM Trans. Math. Softw.* **31**, 363-396 (2005).
