# Supplementary figures and images for: Ballistic diffusion fronts in biomolecular condensates
Source: Nat Nanotechnol. 2025 Jun 6;20(8):1062–70. doi: 10.1038/s41565-025-01941-0 (PMC12373509; doi:10.1038/s41565-025-01941-0)

**a** Uncropped gel for Extended Data Fig. 1b

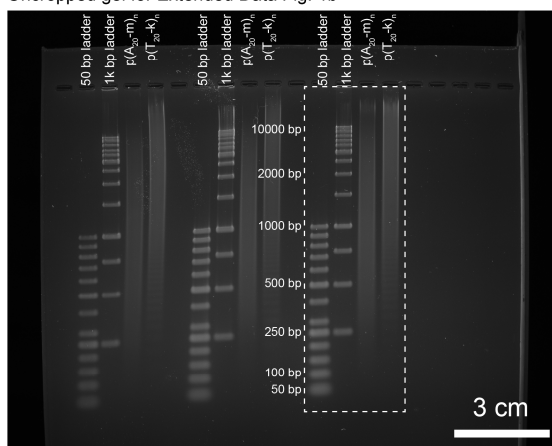

**b** Uncropped gel for Extended Data Fig. 7d

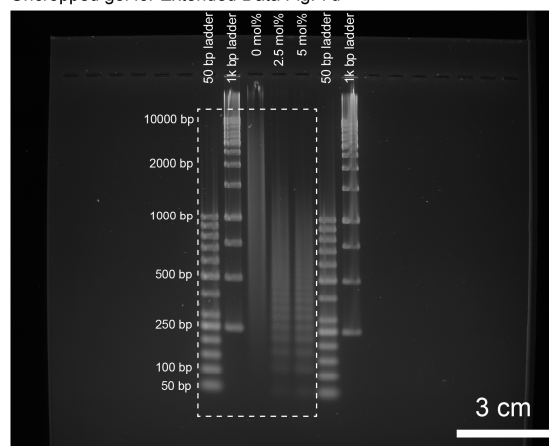

Supplement: Supplementary file 21 — Unprocessed gels. [file 41565_2025_1941_MOESM21_ESM.pdf]
